# Early antiretroviral therapy in SIV-infected rhesus macaques reveals a multiphasic, saturable dynamic accumulation of the rebound competent viral reservoir

**DOI:** 10.1371/journal.ppat.1012135

**Published:** 2024-04-09

**Authors:** Brandon F. Keele, Afam A. Okoye, Christine M. Fennessey, Benjamin Varco-Merth, Taina T. Immonen, Emek Kose, Andrew Conchas, Mykola Pinkevych, Leslie Lipkey, Laura Newman, Agatha Macairan, Marjorie Bosche, William J. Bosche, Brian Berkemeier, Randy Fast, Mike Hull, Kelli Oswald, Rebecca Shoemaker, Lorna Silipino, Robert J. Gorelick, Derick Duell, Alejandra Marenco, William Brantley, Jeremy Smedley, Michael Axthelm, Miles P. Davenport, Jeffrey D. Lifson, Louis J. Picker

**Affiliations:** 1 AIDS and Cancer Virus Program, Frederick National Laboratory for Cancer Research, Frederick, Maryland, United States of America; 2 Vaccine and Gene Therapy Institute and Oregon National Primate Research Center, Oregon Health & Science University, Beaverton, Oregon, United States of America; 3 Infection Analytics Program, Kirby Institute for Infection and Immunity, University of New South Wales, Sydney, Australia; University of Wisconsin, UNITED STATES

## Abstract

The rebound competent viral reservoir (RCVR)–virus that persists during antiretroviral treatment (ART) and can reignite systemic infection when treatment is stopped–is the primary barrier to eradicating HIV. We used time to initiation of ART during primary infection of rhesus macaques (RMs) after intravenous challenge with barcoded SIVmac239 as a means to elucidate the dynamics of RCVR establishment in groups of RMs by creating a multi-log range of pre-ART viral loads and then assessed viral time-to-rebound and reactivation rates resulting from the discontinuation of ART after one year. RMs started on ART on days 3, 4, 5, 6, 7, 9 or 12 post-infection showed a nearly 10-fold difference in pre-ART viral measurements for successive ART-initiation timepoints. Only 1 of 8 RMs initiating ART on days 3 and 4 rebounded after ART interruption despite measurable pre-ART plasma viremia. Rebounding plasma from the 1 rebounding RM contained only a single barcode lineage detected at day 50 post-ART. All RMs starting ART on days 5 and 6 rebounded between 14- and 50-days post-ART with 1–2 rebounding variants each. RMs starting ART on days 7, 9, and 12 had similar time-to-measurable plasma rebound kinetics despite multiple log differences in pre-ART plasma viral load (pVL), with all RMs rebounding between 7- and 16-days post-ART with 3–28 rebounding lineages. Calculated reactivation rates per pre-ART pVL were highest for RMs starting ART on days 5, 6, and 7 after which the rate of accumulation of the RCVR markedly decreased for RMs treated on days 9 and 12, consistent with multiphasic establishment and near saturation of the RCVR within 2 weeks post infection. Taken together, these data highlight the heterogeneity of the RCVR between RMs, the stochastic establishment of the very early RCVR, and the saturability of the RCVR prior to peak viral infection.

## Author summary

Antiretroviral therapy (ART) stops HIV replication without impacting the “rebound-competent viral reservoir” (RCVR), comprised of persistent, already infected cells that can rekindle active HIV infection once ART is stopped. Despite much effort, the characteristics of the clinically relevant RCVR remain poorly defined. We developed a SIV-infected rhesus macaque model and studied temporal development of the RCVR during primary SIV infection, characterizing the impact of RCVR establishment dynamics on post-ART SIV rebound dynamics. Despite measurable SIV viremia, the viral dissemination that developed up to day 4 of SIV infection was highly labile, unable to form an RCVR that can mediate viral rebound after discontinuation of one year of ART. From days 5 to 7, both the extent of primary infection assessed by plasma viremia and post-ART viral reactivation rates exponentially increased in concert, but from day 7 to 12 post-infection, reactivation rates only marginally increased whereas extent of infection continued exponential expansion. This disconnect suggests that the multiphase establishment of the RCVR is saturable and therefore constitutes only a subset of overall SIV infection when ART is initiated after day 7. These observations have important implications for therapeutic targeting of the RCVR and for measuring the impact of such therapies on RCVR size *in vivo*.

## Introduction

The major obstacle to a more definitive treatment of HIV infection that would obviate the need for ongoing antiretroviral therapy (ART) is the rebound competent viral reservoir (RCVR)—virus that persists despite long term suppressive ART and can give rise to recrudescent infection when ART is stopped [1,2]. While currently available antiretroviral drugs effectively block new rounds of infection, they do not impact cells infected prior to treatment initiation. The cells harboring replication competent virus that can persist during ART, including immunologically silent, latently infected cells [3,4] and persistent expanded clones of CD4^+^ T cells, can reactivate viral expression and give rise to renewed systemic infection when treatment is discontinued. Numerous assays have been developed to measure different viral parameters in an effort to characterize the RCVR, including quantitation of different forms of viral DNA, viral RNA, proviral genetic integrity, and various formats of *ex vivo* viral outgrowth assays, each with their respective strengths and limitations [reviewed in [5]]. Despite these assays, the underlying biology of the RCVR remains poorly understood and time to rebound following discontinuation of ART in humans remains the benchmark gold standard for assessing the RCVR and evaluation of candidate interventions intended to reduce or eliminate it [6,7]. In practice, limited dynamic range, extensive interindividual variability, and a predicted requirement for profound impact of an intervention targeting the RCVR in order to demonstrate a convincing beneficial result have limited the practical utility of applying time to rebound as a metric in interventional studies in humans [8]. While considerable experience has been gained in managing the complexities and potential risks of treatment interruption in the setting of clinical research [6,9], use of the approach has not provided fundamental insights into the biology of the RCVR.

Nonhuman primate (NHP) models have been instrumental in providing insights into fundamental aspects of HIV disease. To better understand how the RCVR is established and how this impacts its subsequent biology, we developed a reductionist RM model and associated analytical methods and applied mathematical modeling to study these processes [10,11]. A subsidiary goal of this work was to develop an optimized RM model to evaluate candidate interventions designed to reduce RCVR size. The basis for this model is the barcoded SIVmac239M virus—a synthetic viral swarm that contains thousands of variants each with short barcode sequence stably inserted within the viral genome creating a genetically distinct but phenotypically identical stock [10,11]. Use of this virus model allows seeding of tissues with uniquely barcoded, but otherwise genetically and phenotypically identical variants that can be individually identified by sequence analysis. This model viral swarm has provided important insights in the interrogation of numerous essential aspects of HIV/SIV viral dynamics [10–15].

Here, we used the barcoded SIV-RM model to study the dynamics of RCVR establishment during primary infection by varying the time of ART initiation to generate different levels of RCVR. By initiating infection with a standardized intravenous dose of SIVmac239M and then starting ART at progressively later times thereafter, each successive group of RMs experienced additional replication cycles prior to ART initiation, leading to an increasing amount of viral production and total number of infected cells. After 1 year of treatment, ART was discontinued. Some RMs starting ART at the earliest post-infection times either failed to rebound or showed measurable plasma rebound with only a few rebounding lineages while RMs starting ART later all rebounded with many distinct viral lineages. Time to detectable plasma viremia provided limited resolution for discriminating RCVR size, while calculated viral reactivation rates were variably correlated with the pre-ART plasma viral load (pVL), depending on the timing of ART initiation. These results indicate that the accumulation of an enduring RCVR during primary infection starts slowly with a low initial probability of successful establishment of a durable RCVR, followed by an exponential increase in the RCVR corresponding to the rapid expansion of pVL in primary infection, only to dramatically slow prior to pVL reaching its peak. The findings highlight the stochastic establishment of RCVR, the heterogeneous and dynamic multiphase process of establishment of the RCVR, and the apparent saturability of the RCVR prior to achievement of peak pVL. These previously unappreciated aspects of the RCVR provide basic insights into the establishment of the primary obstacle to more definitive treatment of HIV infection, along with guidance for optimization of NHP studies for evaluation of RCVR targeting strategies.

## Results

### Large differences in viral measurements based on timing of ART initiation

Thirty-five purpose-bred, Indian-origin rhesus macaques (*Macaca mulatta; RM*) were infected intravenously with SIVmac239M [a virus stock containing ~10,000 viral variants of a SIVmac239 infectious molecular clone, each of which contains a unique 34-base “barcode” between the vpx and vpr genes, allowing for genetic tracking of distinct individual, phenotypically equivalent viral lineages over time [10,11]]. Groups of RMs were treated with daily combination ART (DTG/TDF/FTC) [16] starting at 3, 4, 5, 6, 7, 9, or 12 days post infection (dpi) with 5 monkeys per group (henceforth referred to as d3, d4, d5, d6, d7, d9, and d12). We measured multiple virologic parameters to characterize establishment of infection and the impact of treatment initiation at different times.

**Fig 1:**
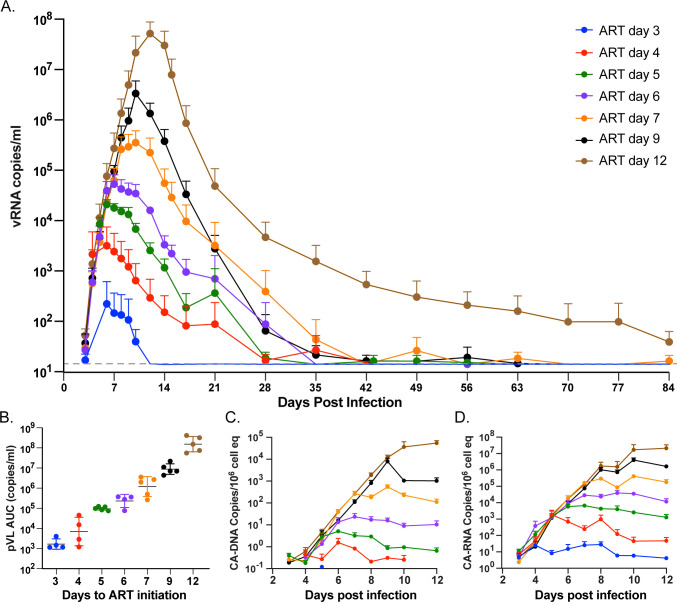
Pre- and early post-ART viral dynamics. Average pVL grouped by time to ART initiation (A). Area-under-curve of total pVL was calculated between infection and day 84 post infection (B). CA-DNA (C) and CA-RNA (D) accumulation between infection and day 12 post infection are shown. Groups are color-coded based on days post infection until ART: d3 (blue), d4 (red), d5 (green), d6 (purple), d7 (orange), d9 (black) and d12 (brown).

Plasma viral RNA rapidly increased during primary infection followed by a rapid decline after ART initiation (**Fig 1A**). As an indicator of systemic innate immune activation following SIV infection [17,18], we determined the induction of CD169 expression on CD14^+^ classical monocytes, a response that has been shown to be upregulated by type 1 interferon and other TLRs triggered by viral infection [19,20]. The frequency and duration of CD169 induction on circulating monocytes tracked with increasing time to ART initiation, with early ART RMs (d3 and d4) showing little to no activation and late ART RMs showing increasing activation response frequencies and time spans commensurate with the duration of unsuppressed infection (**S1 Fig**). Of note, monocyte activation returned to baseline after ~9 days of ART in all RMs. During this time period, the measured peak pVL increased on average 9.1-fold between groups resulting in peak pVLs that spanned nearly 7 logs (**S2 Fig**). After ART initiation, three RMs (A5, B5, D5) failed to fully suppress virus despite daily ART administration. Barcode sequence analysis of on-ART plasma from these RMs revealed the presence of only a single barcode, different in each RM, suggesting virus production from a large infected cellular clone rather than a failure of ART to inhibit ongoing viral replication with infection of previously uninfected cells (**S3 Fig**). Interestingly, virus in one RM (B5) generated known drug resistance mutations including RT: K65R and INT: E92Q [21, 22] sometime after 130 days post ART. All 3 non-fully ART-suppressed RMs were excluded from the study. For the remaining 32 RMs, the pVL area-under-curve (AUC) was calculated from day 0 to 84 when pVL was below 15 copies/ml for most RMs, which revealed an average of 8.2-fold increase in total AUC between groups with a more than 5-log difference between the d3 and d12 groups (**Fig 1B**). In addition to pVL, the timing of ART administration also affected the levels of SIV nucleic acids found in peripheral blood mononuclear cells (PBMCs). Peak PBMC SIV cell-associated (CA)-DNA and CA-RNA increased between successive groups by an average increase of 11.2- and 14.2-fold, respectively (**Figs 1C–1D, S4 and S5**). These initial viral dynamics were consistent with prior results for SIV in RMs with delays in ART initiation increasing all viral measurements by nearly 10-fold per day [18].

### Plasma viral load decay dynamics

**Fig 2:**
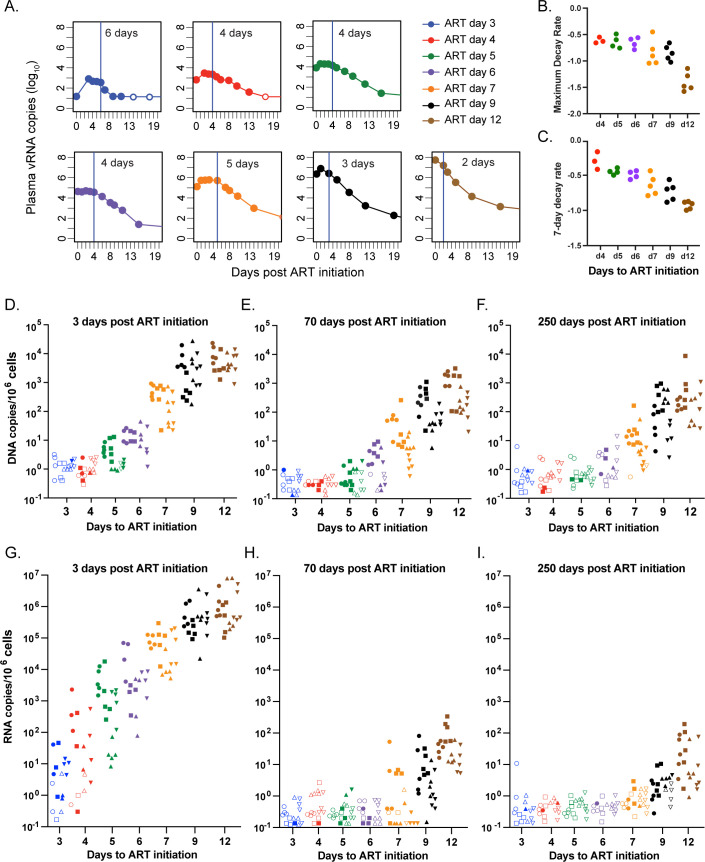
Viral decay dynamics affected by timing of ART initiation. Delay in first phase viral decay following ART initiation (A). The primary phase 1 decay rate using both a maximal decay rate (B) and a 7-day average rate (C) excluding 6 RMs with low pVL where rates could not be estimated. Decay kinetics of CA-DNA (D-F) and CA-RNA (G-I) within PBMC (circle), LNMC (square), DUO (up triangle) and BM (down triangle) from 3-, 70- or 250-days following ART initiation. Unfilled symbols represent samples with no viral signal detected and are plotted at the calculated limit of detection (LOD) for each sample based on specimen input. Groups are color-coded based on days post infection until ART: d3 (blue), d4 (red), d5 (green), d6 (purple), d7 (orange), d9 (black) and d12 (brown).

We next assessed differences in primary pVL decay kinetics across the treatment groups, which span vastly dissimilar pre-treatment total viral burdens. Interestingly, the time between ART initiation and onset of exponential decline was often delayed by multiple days in the RMs that initiated ART earliest (**Figs 2A and S6**). The average delay to the onset of exponential decay in pVL was anywhere from 2- to 8-days after ART initiation. The phenomenon of delay prior to the onset of initial decline can also be identified in animals from a previous study [18] when ART was started prior to peak pVL, but is completely absent when starting ART at peak or set point pVL (**S7 Fig**). Assuming effective blockade of viral replication by ART, extended delays in the onset of viral decay after ART initiation are not typical when ART begins after peak pVL and reveals a difference in either the length of virus production or the half-life of previously infected cells, or delayed clearance of virus from the blood. We calculated the primary phase 1 decay rate using both a maximal decay rate and a 7-day average rate (**Fig 2B and 2C**). In both estimates, once exponential decline begins, the first phase decay rate was highly correlated with the time of ART initiation (R^2^ = 0.81 for maximum decay rate p<10^−7^ and R^2^ = 0.84 for 7-day exponential decay p<10^−8^). It was also clear that the earlier ART was initiated, the quicker pVL progressed to below assay threshold, with fewer blips detected subsequently (**S8 Fig**). By 70 dpi, pVL in all monkeys in groups d3, d4, d5, d6, d7, and d9 had declined to below 15 copies/ml and often remained below detection even using a more sensitive 1 copy/ml assay. However, pVLs for the d12 group were only infrequently below 15 copies/mL during the entire year of ART.

### Cell-associated viral decay

The accumulation and subsequent decline of SIV CA-DNA was also assessed in PBMCs, peripheral lymph node mononuclear cells (LNMCs), duodenal (DUO) and bone marrow (BM) biopsy specimens from day 3, day 70, and day 250 post ART initiation (**Figs 2D–2F and S9A–S9C**). For the d3 and d4 ART groups, there were only a few tissues with detectable CA-DNA across all three time points. For RMs in the d5 and d6 groups, CA-DNA declined 8-fold on average between 3- and 70-days post-ART and only by an average of 2.1-fold between 70- and 250-days post ART. In the d7, d9, and d12 RMs, there was a precipitous decline in CA-DNA between 3- and 70-days post ART with an average of 49-fold decrease across all tissues, which stabilized thereafter with only a 2.4-fold average decline between 70- and 250-days post. Similar viral dynamics were observed for SIV CA-RNA with an even greater decrease following ART administration (**Figs 2G–2I and S9D–S9F**). There was a dramatic decrease in CA-RNA between days 3 and 70 post ART initiation with at least a 4-Log_10_ average reduction across all tissues. For the period between 70- and 250-days post ART, CA-RNA was detected nearly exclusively in RMs from groups d7, d9 and d12, which showed an 8-, 4-, and 6-fold average reduction across all tissues, respectively.

### Pre-ATI infected cell levels

**Fig 3:**
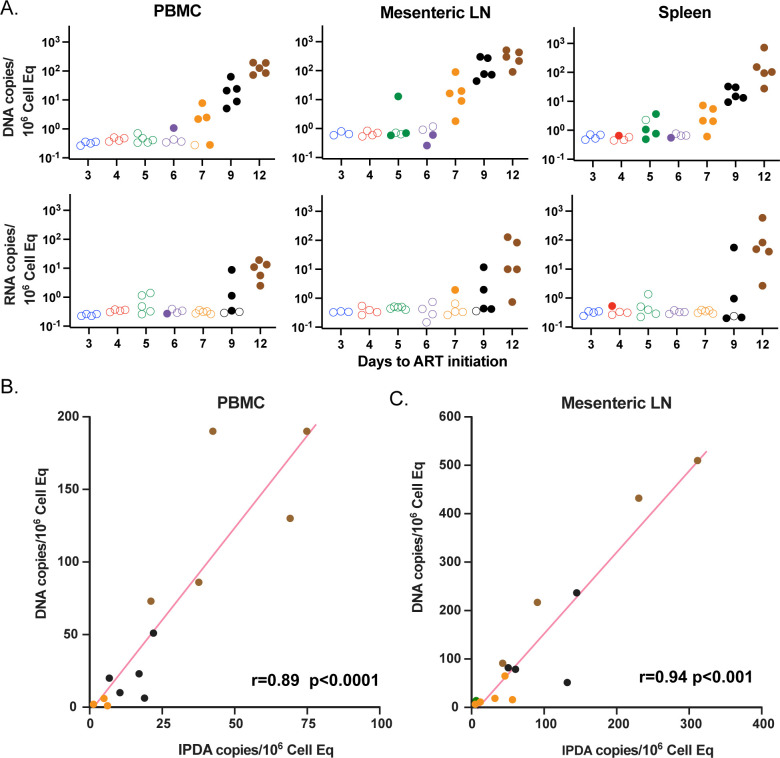
SIV CA-DNA, SIV CA-RNA, and IPDA levels prior to ART interruption using CA-DNA and RNA values and IPDA. Viral RNA and DNA copies/million cells from PBMC, mesenteric LN, and spleen just prior to ART release (A). Unfilled symbols represent samples with no viral signal detected and are plotted at the calculated LOD for each sample based on specimen input. IPDA vs CA-DNA measurements in PBMC (B) and mesenteric LNMCs (C). Groups are color-coded based on days post infection until ART: d3 (blue), d4 (red), d5 (green), d6 (purple), d7 (orange), d9 (black) and d12 (brown).

To assess the extent of persistent infected cell populations just prior to ART interruption (day 350), we utilized a laparoscopic biopsy procedure [23] to sample and quantify SIV CA-DNA and SIV CA-RNA from mesenteric LNMCs and splenic MNCs compared to PBMCs (**Fig 3A**).There were only minimal declines in the frequency of CA-DNA positive PBMC or LNMC specimens from day 250 to pre-ATI (day 350) and modest decreases in levels measured in positive samples (≤ 4-fold), highlighting the slow decline of infected cells over this time range (compare **Figs 2F** and **3A**). Declines in CA-RNA were greater (compare **Figs 2I** and **3A**). Overall, the CA-DNA signal was most prominent within the mesenteric LN specimens. Within the biopsy tissue specimens tested, the majority of detectable CA-DNA after nearly a year of early initiated ART was found almost entirely in the d7, d9, and d12 RMs, while viral RNA positive tissues were found nearly exclusively in d9 and d12 RMs. The qPCR/qRT PCR assays employed in this study target the viral Gag region, which can lead to an overestimation of the number of sequence-intact, presumptively replication-competent viruses in animals treated with late initiation, long duration ART [24]. We compared the gag-based CA-DNA levels to levels determined by intact-proviral DNA assay (IPDA) and we found most genomes to be intact, consistent with our prior finding [25], and significantly correlated in PBMCs (r = 0.89, p<10^−5^) and mesenteric LNMCs (r = 0.94, p<10^−7^) (**Fig 3B and 3C**). Overall, the frequency of SIV RNA and DNA positive cells and tissues, and their corresponding levels associate with time to ART initiation. This model of SIV-RM starting ART at successive days during primary infection after intravenous inoculation generates an extensive, calibrated multi-log range of virus replication and spread prior to ART initiation such that at the time of treatment interruption one year later, at the low end, nearly all tissues and plasma lacked measurable levels of viral DNA and RNA, whereas at the high end, all RMs showed evidence of virus in most tissues and in plasma.

### Viral rebound dynamics following ART release

**Fig 4:**
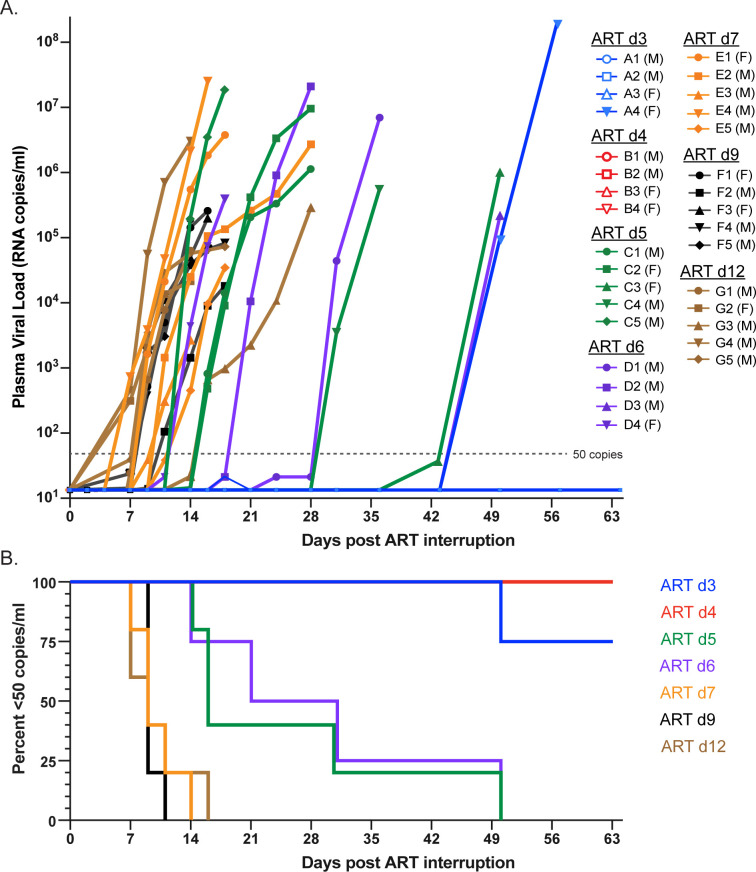
Rebound kinetics and time to detection following ART discontinuation. PVL values starting at treatment interruption through peak rebound (A). Non-rebounding RMs are shown with unfilled symbols and animal sex indicated in legend. Kaplan-Meier plot of time to detectable rebound (50 copies/ml) after treatment interruption (B). Groups are color-coded based on days post infection until ART: d3 (blue), d4 (red), d5 (green), d6 (purple), d7 (orange), d9 (black) and d12 (brown).

The impact of differences in persistent virus levels on viral rebound was assessed following treatment interruption (**Fig 4A**). In the d3 and d4 groups, only a single RM (A4) had measurable rebound viremia (>50 copies/ml) which did not occur until 50 days following treatment interruption. In the d5 and d6 groups, all RMs rebounded between 14- and 50-days. RMs in the d7, d9, and d12 groups rebounded between 7- and 16-days post interruption. Kaplan-Meier analysis of frequency of RMs rebounding (**Fig 4B**) showed significant differences between the median aviremic survival times of RMs starting ART at different days (p<0.0001. Mantel-Cox test), with a linear trend between median survival (no rebound) and time of ART initiation (p<0.0001, logrank test for trend). Pairwise comparison of median survival times (Mantel-Cox test with Benjamini-Hochberg adjustment of p-values for multiple comparisons) revealed clustering of RMs starting ART at d3 and d4 (p = 0.39), another grouping of RMs starting ART at d5 and d6 (p = 0.85), and still another statistically indistinguishable group of RMs starting ART at d7, d9, and d12 (p>0.72). There were however significant differences between RMs starting ART at d3/d4 and d5/d6 (p<0.018), d3/d4 and d7/d9/d12 (p<0.01), and d5/d6 and d7/d9/d12 (p<0.025). Therefore, despite a nearly 1-log increase in pre-ART viral measurements between groups, pre-ART viral measurements were not linearly correlated with time to rebound off ART (r = -0.25, p = 0.25), likely due to limited dynamic range in the time to rebound measurements and inter-animal variability. In the most extreme example, the average difference in pre-ART pVL between d7 and d12 RMs was over 3 logs, yet there was no meaningful difference in time to measurable plasma viremia between these groups.

In the 7 RMs in the d3 and d4 groups that did not show detectable plasma virus rebound within 326 days after ART release, we utilized mAb-mediated depletion of CD8α^+^ cells to facilitate potential rebound of residual virus and followed animals for a minimum of 77 more days. This method transiently depletes CD8α^+^ T cells and NK cells and any associated potential immune inhibition of viral reactivation by such cells, but also induces CD4^+^ memory T cell proliferation, which is in large part due to depletion of CD8α^+^ NK and T cells that utilize IL-15, resulting in increased IL-15 stimulation of CD4^+^ T cells [26, 27]. Anti-CD8α administration was performed three times over a 6-week period but did not induce detectable viral replication detectable in plasma (>15 copies/ml) (**S10 Fig**). Therefore, the virus in all but one RM from the d3 or d4 groups appears to have failed to establish a RCVR capable of persisting beyond one year on ART. Remarkably, this apparent failure to rebound occurred in RM reaching viremia levels of nearly 10^4^ copies/ml in blood at the time of ART initiation. These results are consistent with prior data indicating that in RMs starting ART at 3–4 days post-infection, persistent infectious virus can be demonstrated by adoptive transfer of lymphocytes to a naïve host at 6 months, but not after one year [18]. Together, these results highlight that following early ART initiation, the RCVR is stochastic in its formation and labile in its persistence such that after 400 days off ART most animals, but not all, fail to rebound even after CD8α depletion.

### Barcode sequencing to enumerate rebounding viral lineages

We next analyzed the viral barcodes present throughout the duration of the study to gain a higher resolution assessment of the RCVR that was established when ART was initiated at different times post-infection. A dose of 500 infectious units (IU) (as determined by TZMbl assay) was used to guarantee infection in all RM while minimizing inoculum dose impact on host versus viral dynamics [11]. At this dose, the average number of detectable barcodes in plasma pre-ART was 67 (range 11–135; n = 28) (**S11 Fig**). The barcode distribution in PBMC, BAL, LNMCs, DUO, and BM prior to ART mirrored plasma with 98.7% of all barcodes detected in tissues also identified in blood (**S12 Fig**). Therefore, distinct barcoded lineages are well distributed between RMs and across infected cells in various tissues within and between RMs.

**Fig 5:**
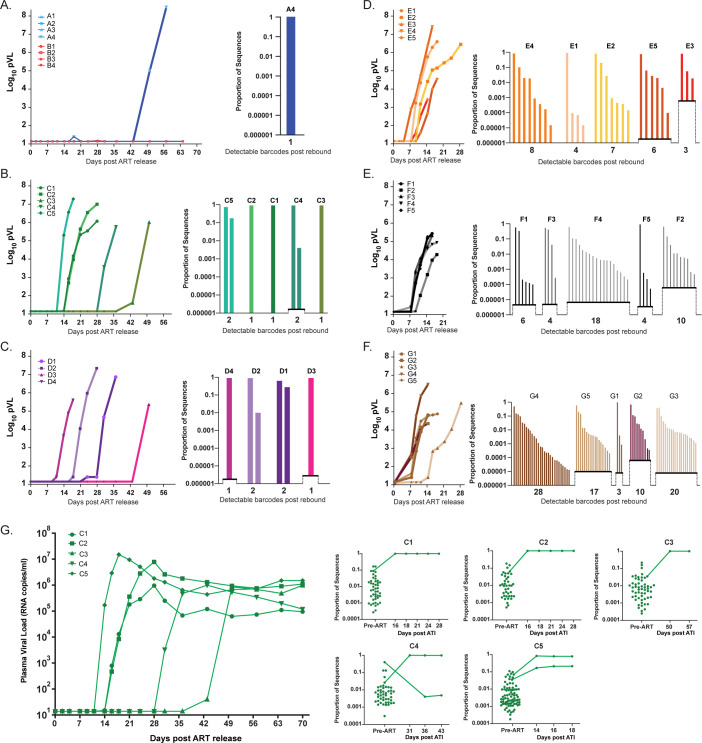
Barcode assessment post rebound. Rebound kinetics and barcodes for each rebounding RM are presented based on the post-infection day ART was initiated with the rebound pVL of each RM shown on the left and the relative barcode proportion at peak rebound shown on the right (A-F). Representative (d5 ART group) of barcode comparisons across time post rebound and compared to pre-ART population (G). LOD is determined by viral input levels and is shown as elevated x-axis levels. Groups are color-coded based on days post infection until ART: d3 (blue), d4 (red), d5 (green), d6 (purple), d7 (orange), d9 (black) and d12 (brown).

Following treatment interruption, barcode sequencing was performed on rebounding virus in plasma. Plasma from the single d3 RM (A4) that rebounded 50 days post-ART release was found to contain only a single viral barcode lineage (**Fig 5A**). Interestingly, there was nothing distinctive about this RM across the virologic parameters measured prior to rebound that distinguished it from the other RMs from the d3 or d4 group that did not rebound (**S13 Fig**). For d5 and d6 RMs, barcode sequencing identified only one or two rebounding lineages per RM following treatment interruption (**Fig 5B and 5C**). This contrasts to the d7, d9 and d12 RMs where the number of detectable rebounding barcodes ranged from 3–8, 4–18 and 3–28, respectively (**Fig 5D–5F**). We sequenced 1 to 4 additional post-rebound plasma time points for each RM (**Figs 5G** and **S14**) and found highly consistent results in each sample with additional rebounding lineages accumulating over time as reactivation events occurred with sufficient levels of replication to be detectable in plasma. We discovered that 50% of viral lineages present in rebound viremia were present in the top 10% of the pre-ART plasma barcode distribution and 80% in the top quartile.

### Reactivation rate measurements extend the dynamic range of detecting differences in RCVR size

**Fig 6:**
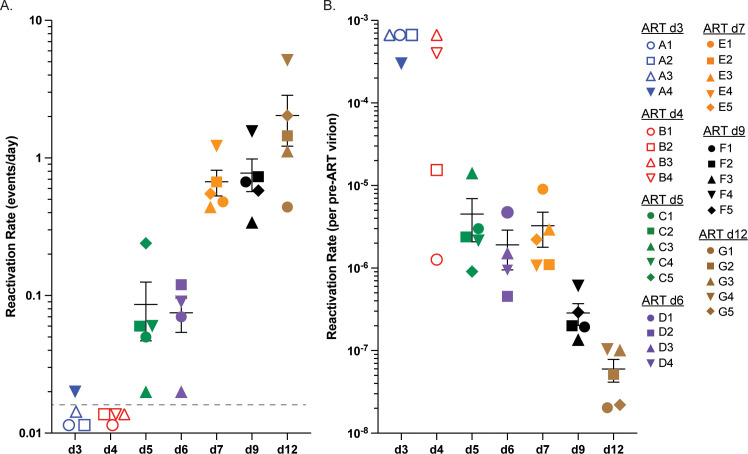
Reactivation rates. Reactivation rates (events per day) of individual RMs within each treatment group are shown with RM-specific symbols (A). Reactivation rates from non-rebounding RMs are shown with open symbol set at LOD of 0.01 events/day. The pre-ART per virion reactivation rate is plotted for each individual RM using LOD and open symbols for RMs that did not rebound (B). No significant differences were found between RMs in group 5, 6 and 7 (p>0.4), however they were each significantly different from groups d9 and d12 (p<0.05), and the latter two groups were also significantly different from each other (p = 0.016). The average rate of d5, d6, and d7 was significantly different from the d9 RMs (12-fold, p<0.001) and d12 RMs (74-fold, p<0.001). Groups are color-coded based on days post infection until ART: d3 (blue), d4 (red), d5 (green), d6 (purple), d7 (orange), d9 (black) and d12 (brown).

Viral reactivation rate (RR) is the average time infected cells activate leading to the production of virus that can successfully progress to measurable systemic viremia. Distinct reactivation events are identified by the number of individual barcode lineages detected in plasma during the window of time between ART release and peak rebound viremia providing a greater resolution in assessing the RCVR than simply time to measurable pVL levels [10]. Because some RM rebounded with only one or two barcoded lineages, the RR for this study was calculated by dividing the number of detectable viral lineages by the total estimated time off ART during which reactivating lineages could grow to detectable levels. In the d3 and d4 groups, only one RM rebounded, with a single barcoded lineage detected, yielding an estimated RR of 0.02 (1 event in 50 days) (**Fig 6A**). The average per group RR for RMs starting ART at days 5 and 6 was not statistically different (0.09 and 0.08, respectively). The RR increased to 0.67, 0.78 and 2.04, respectively, for groups 7, 9 and 12 which was statistically different between groups (p = 0.0016; Kruskal-Wallis).

We next compared these RR to measured virologic parameters within the cohort to see which variables, if any, correlated with or predicted RCVR size at treatment interruption, as assessed by RR. Although RR was correlated with many pre-ART and on-ART viral measurements, it was best correlated with peak pVL at the time of ART initiation (Pearson correlation; r = 0.90, p<10^−11^). Importantly, the RCVR size, as determined by RR, did not accumulate proportionately to viral replication over the entire pre-ART period. We calculated the pre-ART per virion RR by dividing the calculated RR by the corresponding pre-ART peak pVL (**Fig 6B**). If the RCVR increased uniformly at the same rate as the pre-ART viral load, we would expect this ratio to remain constant between treatment groups. However, the reactivation rate per pre-ART pVL differed significantly across groups (p = 0.001, Kruskal-Wallis). While groups d5, d6 and d7 were not significantly different from each other (p> = 0.45), they were each significantly different from groups d9 and d12 (p<0.05), and the latter two groups were also significantly different from each other (p = 0.016; Wilcoxon rank-sum test, Benjamini-Hochberg adjusted). The pre-ART per virion reactivation rate was 12-fold lower for d9 RMs and 74-fold lower for d12 ART RMs compared to group d5, d6, and d7 RMs on average. Furthermore, two RMs (B1 and B2) from the d4 group had pre-ART pVL levels sufficiently high to suggest that they would likely rebound in the 400 days post-ART follow-up but failed to do so. By comparing the virologic measures of RM B1 and B2 against the d5 group where all RMs rebounded, we found only slightly lower pVL, CA-DNA and CA-RNA levels across all time points and in all tissues sampled (**S15 Fig**). These results emphasize the stochastic nature of early development of the RCVR and a lag in the establishment of the RCVR relative to viral replication (d3 and d4 RMs).

**Fig 7:**
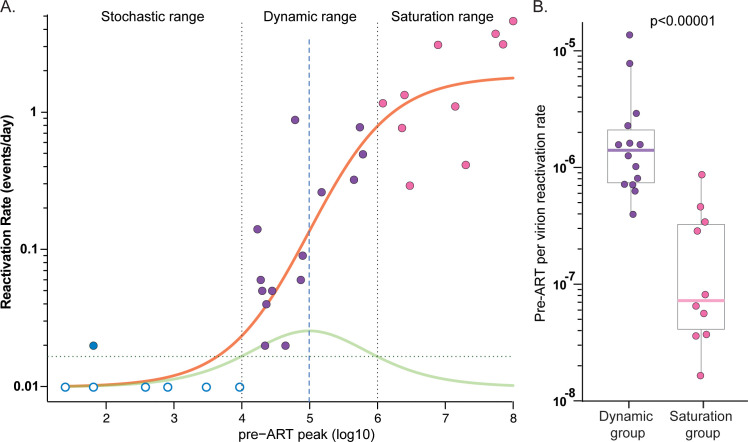
Phases of the accumulation of RCVR during primary infection. The reactivation rate is plotted against pre-ART peak pVL which exhibits a logistic fit (orange curve) (A). The RMs with pre-ART peak VL within the range 4–6 Log_10_ are in the *dynamic range* (purple symbols). The rate of change of the best fitting logistic model is shown by green curve. RMs with pVL <4 Log_10_ (blue symbols) only rarely rebound and fall within the *stochastic range*, while those with >6 Log_10_ peak pre-ART pVL fall within the *saturation range* (pink symbols) where the RCVR accumulation slows. Non-rebounding RMs are shown with unfilled symbols. Comparison of the pre-ART per virion reactivation rate of RMs in the *dynamic range* (purple) versus the *saturation range* (pink) show that RMs in the *dynamic range* have significantly higher pre-ART per virion reactivation rate than those in saturation group (p<0.00001) (B).

To interrogate RCVR accumulation as a function of viral replication, we first plotted RR (log10) against the pre-ART peak pVL (log10) for each of the 32 RMs and assessed whether the data were better described by a logistic model with saturated growth rather than simple linear regression **(Fig 7A)**. The logistic model provided a significantly better fit to the data than the linear regression model based on Akaike information criterion (AIC) scores of 40.14 and 65.92, respectively, with a relative likelihood of 10^−6^ for the linear regression model. We defined the *dynamic range* as the region of pre-ART peak pVLs (10^4^ to 10^6^). This region includes RMs in groups d5, d6 and d7. The *stochastic range* includes the d3 and d4 RMs which had pre-ART peak pVL less than 4 logs where rebound is unlikely to occur after 1 year of ART. The *saturation range* includes d9 and d12 RMs with pre-ART peak pVL higher than 6 logs. For RMs within the dynamic range, a 10-fold increase in peak pVL yielded a 7.6-fold increase in RR (p = 0.002; linear regression), whereas the corresponding increase in RR was only 3.6-fold for RMs within the saturation range (p = 0.022; linear regression). Overall, the median pre-ART per virion RR was over 100-fold higher for RMs within the dynamic range than for those in the saturation range (-5.7 log10 vs -6.9 log10; p<10^−5^; Wilcoxon-test) (**Fig 7B**).

## Discussion

The RCVR is the primary obstacle to more definitive treatment of HIV infection that would obviate the need for continuous ART, and numerous approaches for reducing or eliminating the RCVR have been explored. However, the underlying biology of the RCVR is incompletely understood and the gold standard readout for evaluating RCVR targeted interventions, impact on time to plasma virus rebound following discontinuation of ART, suffers from extensive inter-individual variability and a limited effective dynamic range. Various other indirect virologic measurements have been used to assess treatment impacts on the RCVR, with limited success. To address this situation, we sought to advance two key objectives of HIV cure research. The first was to better understand the dynamic process of establishing and maintaining the RCVR and define how the dynamics of RCVR establishment influence post-ART viral rebound. The second was to develop a NHP model wherein promising RCVR targeting strategies could be tested with the greatest likelihood of obtaining measured reductions in RCVR which might provide useful pre-clinical data to evaluate and optimize various approaches.

The earliest and most dynamic aspects of RCVR establishment are challenging if not impossible to measure in primary HIV infection, especially in tissues, yet it is precisely this time when initiation of ART is the most beneficial in slowing disease progression, preserving or restoring gut barrier integrity, maintaining immune function, and reducing transmission [28]. To address this challenge, we developed a model with intravenous infection of RM using a barcoded SIV in which initiation of ART at different early times after infection results in establishment of an extensive multi-log range of viral seeding of tissues, a portion of which becomes the RCVR. We used quantitative virologic assays and calculated viral reactivation rates incorporating time to post-ATI rebound to characterize establishment and persistence of the RCVR. We employed the RM model to establish that the early establishment of a durable RCVR in the first days following infection is stochastic and/or labile; followed by an exponential phase, which then appears to saturate prior to achievement of peak plasma viremia, with later accumulation of infected cells at and after peak pVL contributing less to the overall RCVR magnitude. These findings provide new basic insights into the biology of RCVR establishment and guidance for optimization of testing of RCVR targeting interventions in NHP.

### Early vs. later ART initiation and duration of ART

Here and previously [18], we showed that a stable, long-lived RCVR was not immediately established upon infection and indeed, was only durably established after 5 days post-infection. These data contrast with Whitney et al [29] where rebound occurred in RMs starting ART at day 1 (1 of 5 RMs), day 2 (3 of 5 RMs) and day 3 (5 of 5 RMs). Two significant differences between these studies include (i) the route of infection (intrarectal versus intravenous) where mucosal infections could conceivably lead to infection of a particular cell type not infected via intravenous inoculation that is more likely to allow establishment of a RCVR and (ii) the time on ART before discontinuation (6 months versus 1 year) which could allow for rebound from a short-lived RCVR, but not a long-lived one. Of note, supporting the latter possibility, we previously observed that infection could be adoptively transferred from RMs after only 6 months of early initiated ART, but not after 1 year on ART [18].

### Unanticipated characteristics in RCVR establishment

Several aspects of the observed early accumulation dynamics of the RCVR in the present study were unexpected, but provide insights into the origins and nature of the RCVR and highlight the advantages of using an NHP model to test novel intervention strategies that seek to reduce the size of the RCVR. These unexpected observations include a delay in the exponential decline of plasma virus in early treated RMs, the stochastic nature of early RCVR establishment, heterogeneity of the size of RCVR, and the multiphasic dynamics and apparent saturability of RCVR establishment, along with a lack of full viral suppression in 3 RMs starting ART in early primary infection, most likely due to early development of expanded clones of infected CD4^+^ T cells.

### Delay in onset of exponential decline of pVL

We observed a delay in the start of exponential decay in RMs starting ART at the earliest times post infection (**Figs 2A–2C, S6 and S7**). We propose that during the earliest days of infection, the infected population is highly skewed towards the more abundant resting target cells that may be initially less productive, but longer lived and able to produce virus for longer periods of time than more activated cells. At peak pVL and beyond, most of the newly infected cells are activated when they are infected, as a consequence of the immunoinflammatory milieu created by the response to the initial infection (**S1 Fig**) [30–33], and therefore likely short-lived, creating the canonical dramatic first phase decline in plasma viral load. Given the extremely low pVL levels from early treated RMs compared to the d12 group and the multi-log increase in the numbers of infected cells during these early pre-ART days, it is remarkable that the earlier treatment begins, the longer the delay before first phase decay begins, the slower that decay is, and in some instances as described, results in a complete failure to suppress virus (**S3 Fig**). Our results are consistent with persistent expanded CD4^+^ T cell clones harboring proviruses that can be established early in infection [34] and can contribute to persistent residual viremia on fully suppressive ART.

### Stochasticity in establishment of the RCVR

We postulate that quantitative and qualitative characteristics of the cell populations infected early after viral challenge, including their capacity for clonal expansion, influence the success or failure of RCVR establishment. We found that only one RM out of 8 rebounded after 1 year of ART when starting ART at day 3 or 4 (**Fig 5**). Strikingly, starting ART just one day later, all RMs rebounded with one or two distinct viral lineages. In the single RM that rebounded from the day 3 group, there were no obvious virologic measurements that seemed to potentially explain the establishment of a RCVR in this RM and not the others, many of which had much higher pre-ART viral measurements (**S13 Fig**). Rebound in RM A4 may be due to clonal expansion of antigen-specific cells with a higher-than-average chance of reactivation. Furthermore, two RMs treated at day 4 had very similar but slightly lower virologic measures than day 5 ART RMs (**S15 Fig**), yet did not rebound. These data suggest that when pre-ART pVL is below ~10,000 copies/ml, establishment of the RCVR is sufficiently variable to prevent rebound within 400 days off-ART in most, but not all animals. This viral load level appears to define the threshold level of pre-ART replication required (in this model system) for consistent, successful rebound, below which either a RCVR that persisted until ATI was not established at all, or the established RCVR was too small for stochastic reactivation to occur within 400 days (even with CD8α depletion). These data clearly indicate that establishment of a spreading SIV infection in and of itself is insufficient to produce a long-lived RCVR, and that such a long-lived RCVR requires that infection penetrate target cell populations that are either long-lived themselves or are capable of clonal expansion to maintain the infected cell lineage on long-term ART. The anatomic and cellular nature of this RCVR (infected cell) subset remains speculative, but it could represent an intrinsically distinct population of CD4^+^ T cells or alternatively changes in CD4^+^ T cell population due to inflammation, massive CD4^+^ T cell depletion, and the onset of adaptive immunity that affect the potential for infected CD4^+^ T cells to persist.

### Heterogeneity in time to viral rebound

In addition to the stochastic nature of the formation of the RCVR in early treated RMs, we also found heterogeneity in the time to rebound, the number of rebounding lineages, and reactivation rates between RMs within the same day of ART initiation group. We sought to generate a NHP model system that would be highly reproducible and consistent within treatment groups by using a clonal virus challenge model, a high-dose intravenous challenge model, and using 5 RMs per group, but were only partly successful in this. This model did indeed provide consistent viral measurements during primary infection and time to suppression. Plasma viral load measures, AUC, and cellular viral RNA and DNA levels were highly similar within each group (**Figs 1, 2, 3, S2, S4, S5 and S8**). Furthermore, the magnitude of change between groups in viral CA-RNA and CA-DNA measures was also consistent (**Figs 1, 2, 3, S2, S4, S5 and S8**). However, the absolute RCVR sizes differed between RMs within the same groups which could be due to the RCVR size at the time of ART initiation or due to the natural underlying changes in cells harboring infectious virus with waxing and waning of clonally expanded populations [35]. Additionally, there is variability in the reactivation from latency and virus production and spread which affects the time to detection and the number of detectable barcode lineages when rebound occurs but is based on only a few rebounding events (**Fig 4**). Furthermore, the proportional differences between barcodes can be considerably different between RMs with the same number of rebounding lineages, reflecting underlying stochasticity in the time of initial reactivation and subsequent early expansion of these lineages conferring a “founder effect” competitive advantage. Additionally, the number of detectable post-ATI barcodes in later treated RMs varied widely within each group, up to nearly 10-fold (**Fig 5**). While reactivation rates are thus also variable, similar to other viral measurements, they are the most consistent measure of RCVR size and by accounting for the pre-ART viral load level within the estimated RR, we were able to define more consistent rebound kinetics in relation to time of ART initiation. Therefore, given the inherent heterogeneity that the biology of the RCVR entails, generating a highly homogeneous RCVR using a virus model that allows for reactivation rate estimates provides important advantages for understanding the basic biology of establishment and persistence of the RCVR, as well as in assessment of novel strategies intended to reduce or eliminate it.

### Lability of early infected cells

The ability of infected cells to persist despite fully suppressive ART administration is a defining property of the RCVR. In this study, only one of 8 RMs starting ART within 4 days of infection rebounded within 400 days post ART release. This failure to rebound suggest that in this model an RCVR capable of supporting after 1 year of ART is not established until after 4 days of infection and pVLs >1x10^4^ copies/mL. As discussed above, we hypothesize that primary SIV infection must penetrate and establish latency in a long-lived and/or expandable cell population to achieve a RCVR that can mediate rebound 1 year later. There are several lines of evidence, including findings in this study, that support the labile nature of the persistence of early infected cells during ART. This includes the four animals in the day 3/4 ART initiation groups that failed to rebound despite pre-ART pVL as much as 2 logs higher than RM A4 which did rebound. Presumptive quantitative or qualitative changes in the early infected cells in RM A4 that enabled eventual rebound and inverse changes in the other animals with higher pre-ART pVL levels that failed to rebound suggest dramatic changes in these SIV-infected cell populations are possible. Furthermore, it is reasonable to think that the well documented waxing and waning of the expanded clones of infected CD4^+^ T cells [35] may have a greater impact when fewer cells are infected due to the higher potential for stochastic extinction at low population sizes, and conversely, the higher potential of clonal expansion of limited individual lineages to increase the absolute RCVR size (e.g. RM A4). Additionally, we showed previously in RMs treated similarly to this study, that 6 months of ART allowed for consistent viral outgrowth, but 1 year on ART prevented rebound in these very early treated animals [18], confirming that the persistence of early infected cells able to mediate viremic rebound is variable. Therefore, given the potential for expansion or decay of virally infected cell populations due to natural cellular dynamics of clonally expanded cells, it is important that experiments evaluating targeted interventions to reduce or eliminate the RCVR include appropriate comparative control groups and maintain animals on ART for at least 1 year to control for spontaneous clearance of infected cells.

### Saturability of the RCVR

Among the several important implications of these results for understanding the biology of RCVR establishment, possibly the most significant are the parameters that define the early dynamics of RCVR establishment, for which we demonstrate three sequential phases, as assessed by timing of ART initiation to prevent further seeding of the RCVR. The *stochastic phase* encompasses the first several days of infection where RMs with pVLs from 25 to nearly 10,000 copies at ART initiation only rarely reactivate and produce rebound viremia when ART is discontinued after a year of treatment (**Fig 7**). In the *exponential phase*, animals have pre-ART pVL between 10^4^and 10^6^, and this is the time when the RCVR is expanding in parallel with exponential increases in pVL such that a 10-fold increase in peak pVL yielded a 7.6-fold increase in RR. In the subsequent *saturation phase*, the accumulation of the RCVR slows significantly with most of the replicating virus, reflected in the pVL and accompanying increases in viral CA-DNA in PBMC and tissues, corresponding to short-lived cells that do not contribute to the durable RCVR, leading to divergence between continued increases viral CA-DNA levels and slowed accumulation of the RCVR. This *saturation phase* begins at 6 log_10_ pVL and extends through peak viremia. Our data thus indicate that the initial burst of replication in primary infection though 6 log_10_ pVL (~day 7 in this model) can lead to a RCVR that is capable of lasting at least one year on ART. After that point, increasing viral replication does not proportionately further increase the RCVR size. It remains possible that at later timepoints of ART initiation, the early established long-lived infected cells may be replaced by cells infected with more recently replicating virus, without a meaningful increase in RCVR size [36]. As alluded to above, the transition from infections with an unstable infected cell population to infections with a RCVR capable of persisting through at least one year of ART, and finally to infections in which the RCVR becomes effectively saturated, likely stem from differences in the size of the infected cell population in concert with the longevity and persistence of the infected cells and viral seeding of cells capable of persisting and/or clonally expanding on ART, which comprise only a subset of the total infected cells. In the same way that failure to establish a durable RCVR might reflect insufficient infection levels to successfully penetrate the presumptive persistent subset of target cells, saturation of the RCVR might reflect a limited size of this long-lived population. Alternatively, dynamic changes in later stages of infection–e.g., levels of inflammation, massive CD4^+^ T cell depletion, the onset of adaptive immunity–that change CD4^+^ T cell turnover and homeostasis, may limit further net increases in the RCVR. In the most general sense, these data indicate that the RCVR is not solely determined by the overall quantitative level of infection, but rather, is likely also dependent on qualitative aspects of the infected cells, including the degree of infection penetrance into long-lived cells and/or the degree to which infected cells can undergo clonal expansion. These dynamic processes involved in RCVR establishment and saturation increase the heterogeneity of the NHP model and must be accounted for when developing NHP cure models for evaluation of RCVR targeting interventions but can be exploited to increase the dynamic range of readouts to assess impacts on the RCVR.

### Lack of full suppression

When ART was initiated at peak pVL (d12 group), we found that while all 5 animals were suppressed to below 50 copies/ml, only 1 RM reached below 1 copy/ml highlighting the slow decline to reach full suppression. The likely cause of this low-level viremia is virus production from long-lived, previously infected cells, including expanded clones and not viral replication [4, 37–39]. While this type of delay to full suppression dynamics in high pVL animals has been reported [16], it was surprising to document complete drug failure in three RMs receiving the well-established three drug ART regimen (DTG/TDF/FTC). Strikingly, this was observed in RMs with low pVL that started ART early (3, 4 and 6 dpi). Sequence analysis revealed residual plasma viremia on ART was due in each case to a single (but different) barcoded virus lineage in each of the 3 animals (**S3 Fig**). Although the current study did not include confirmatory integration site sequencing, we posit that the most plausible explanation for this residual non-suppressible viremia is that it reflects the cumulative virus production from an early established and clonally expanded infected CD4^+^ T cell population [34,40]. Interestingly, the virus in the remaining RM generated known drug resistance mutations [21,22] which caused a complete failure to suppress. Due to sampling limitation, the exact timing of resistance could not be determined, but many months of continuous production of infectious virus allowed for some viral replication and selection of drug resistance in one RM with a sustained pVL of ~1,000 copies/ml while on ART. At treatment interruption, the clonally expanded barcode in each RM was the source of rebounding virus, confirming that this clone dominated the viral population and produced replication-competent viruses. We conclude that virus infection followed by extensive cellular proliferation resulted in a clonal, virus-producing population that was the cause of this non-suppression and highlights the occurrence and importance of establishment and eventual expansion of clonal populations of infected CD4^+^ T cells, beginning during the earliest events post-infection and capable of contributing to the stable, long-lived, RCVR.

We describe here a model of barcoded SIV infection in RMs with specific viral inoculum dose and timing of ART initiation designed to generate an extensive dynamic range in RCVR seeding. NHP models for HIV infection have provided foundational insights into viral transmission and pathogenesis and have enabled preclinical evaluation of vaccine and non-vaccine prevention approaches, new treatment agents and modalities and RCVR targeting interventions. At a practical level, this study sought to develop an approach where the size of the RCVR is normalized in a calibrated range, optimal for evaluation of RCVR targeting interventions and able to provide greater accuracy and dynamic range than simple measurements of time to measurable rebound viremia. Balancing biology, practical and logistical considerations, including budgetary constraints, with concern for animal welfare, we designed the approach for an *in vivo* phase of approximately 1 year.

There are multiple indirect assays available that have been used to try to predict the impact of RCVR targeted interventions on viral rebound, using various measured virological parameters but all have limitations [5]. Therefore, most NHP studies for evaluation of RCVR targeted interventions are limited to treatment interruption phases where time to measurable off ART viremia is the only or the major measured output of the study. It is becoming increasingly appreciated that “off ART time to measurable rebound viremia” is a poor measure of treatment efficacy in both large and small RCVR models because meaningful reductions in the size of the RCVR are not routinely demonstrable using only this parameter [8]. For low RCVR size studies where infrequent stochastic reactivation is at play, reductions in the RCVR size will only be discernable with prohibitively large group sizes, whereas when the RCVR is large, even potentially large reductions in the RCVR might not be detectable in the likely event that at least one reactivation event occurs soon after ART withdrawal.

### Conclusions

The barcoded virus model system provides additional analytical resolution compared to time to detectable plasma rebound viremia by measuring how many total viral lineages contribute to viral reactivation and rebound. Like time to rebound, reactivation rate assessments also have limitations in dynamic range to successfully measure distinct events. While the stochastic development of the RCVR and the stochastic nature of rebound itself both constrain the usefulness of time to detection of measurable rebound viremia as a readout parameter, a barcoded virus model with early ART initiation can be used to define an optimal dynamic window to detect intervention dependent reductions in the RCVR while also providing increased resolution in assessing the rebounding population. The *in vivo* phase of this study design can be completed within one year providing a comparatively inexpensive and efficient model to test novel RCVR targeted intervention strategies, with inclusion of appropriate control groups. Information gleaned from this model should be highly applicable to people who started ART immediately after initial HIV diagnosis during primary infection. These individuals often retain a greater intact immune capacity and have a smaller RCVR, making them the most likely candidates to respond to RCVR reducing treatments. If a particular intervention provides promising data in this early ART model, further validation of the intervention may be obtained using a NHP model system where ART is started in chronic infection and treatment is maintained for up to 3 years [41]. Although an eradicative cure strategy may not be achievable when an intervention induces only small changes to the RCVR, the model system presented here provides the capacity to detect even modest changes and thus affords the opportunity to optimize and quickly retest new interventions, alone and in combination, for feasibility and effectiveness.

The model presented here is a reductionist one, that explicitly does not account, by design, for numerous clinically relevant complexities in people living with HIV that would represent potentially confounding influences in the NHP model. These include use of a clonal barcoded virus that does not represent the potential sequence and phenotypic heterogeneity of HIV variants [10], early initiation of ART and limitation of treatment to 1 year that minimizes the impact of adaptive immune responses [42], while retaining intact viral genomes due to early ART initiation [25], and the use of a highly neutralization resistant challenge virus [43] that limits the contribution of autologous neutralizing antibody responses to viral rebound [44, 45]. While acknowledging these complexities, the model design that excludes these factors allows focus on the essential characteristics of viral factors in establishment of the RCVR, with implications for capacity for viral rebound when ART is discontinued. In so doing, it has provided insights into the biology of the RCVR and guidance for design of optimized experiments to evaluate candidate RCVR targeting interventions.

## Materials and methods

### Ethics statement

All studies were conducted with the approval of the Oregon National Primate Research Center’s Animal Care and Use Committee (TR03_IP00001053), under the standards of the US National Institutes of Health Guide for the Care and Use of Laboratory Animals.

### Rhesus macaques

This study used a total of 35 purpose-bred rhesus macaques (*Macaca mulatta*) of Indian genetic background comprised of 23 males and 12 females distributed evenly between groups. One additional RM was chronically infected with SIVmac239 and used as positive control for CD8α depletion. Animals were specific pathogen free (cercopithecine herpesvirus 1, D-type simian retrovirus, simian T-lymphotropic virus type 1, rhesus rhadinovirus, and Mycobacterium tuberculosis) prior to study initiation and screened for common Mamu alleles (Mamu-A*01/-A*02 and Mamu-B*08/-B*17) using sequence-specific priming PCR as previously described [46]. Six RMs were A*01 (B4, C1, D3, E3, E5, and G4), two were B*17 (F3 and G1), and one was B*08/B*17 (F2). Daily ART consisted of subcutaneous injections of 5.1 mg/kg tenofovir disoproxil (TDF), 40 mg/kg emtricitabine (FTC) and 2.5 mg/kg dolutegravir (DTG) combined in a solution containing 15% (v/v) kleptose at pH 4.2, as previously described [16].

### CD8^+^ T Cell Depletion

RMs without measurable plasma viremia after ART interruption and one control RM were treated with anti-CD8α mAb, MT807R1 (Nonhuman Primate Reagent Resource), administered SQ at 50 mg/kg on 326 days post-ATI then IV at 50 mg/kg on 340 and again on 354 days post-ATI. This treatment typically results in depletion of >99% of circulating CD8α^+^ T and NK cells [47].

### Immunophenotyping

Phenotype of leukocyte populations in blood were characterized by flow cytometry, with whole blood stained as previously described [18]. Polychromatic (8–14 parameter) flow-cytometric analysis was performed on an LSR II BD instrument using Pacific blue, BUV395, BUV495, BUV737, BUV805, BV421, BV510, BV570, BV605, BV650, BV711, BV786, FITC, PE, PE-Texas red (PE-CF594), PE-Cy7, PerCP-Cy5.5, APC, APC-Cy7, and Alexa 700 as the available fluorescent parameters. Combinations of fluorochrome-conjugated monoclonal antibodies used for staining included anti-CD3 (APC-Cy7; BD Biosciences, Custom Bulk 624072), anti-CD4 (L200: BV786; BD Biosciences, Custom Bulk 624159), anti-CD8α (DK25: Pacific Blue; SK1: BUV737), anti-CD11c (3.9: APC; Life Technologies, Custom Bulk CUST01764), anti-CD14 (M5E2: FITC; BioLegend, Custom Bulk 94202), anti-CD16 (3G8: BV650; BD Biosciences, Custom Bulk 93384), anti-HLA-DR (L243: PE-DAZZ; BioLegend, Custom Bulk 93957), CD20 (2H7: Alexa Fluor 700; BioLegend, 98488), anti-CD40 (5C3: BV510; BD Biosciences, Custom Bulk 624146), anti-CD80 (L307.4: BUV; BD Biosciences, Custom Bulk 624309), anti-CD86 (2331 [FUN-1]: BV711; BD Biosciences, Custom Bulk 624316), anti-CD123 (7G3: PerCP-Cy5.5; BD Biosciences, Custom Bulk 624060) and anti-CD169 (7–239: PE; BioLegend, Custom Bulk 346004). Instrument setup and data acquisition procedures were performed as previously described [26,47,48]. List mode multiparameter data files were analyzed using the FlowJo software program (BD Biosciences). Myeloid cells were defined as CD3-, CD20-, CD8α-, HLA-DR+ mononuclear cells and subcategorized by expression of CD14 and CD16 as classical monocytes (CD14+CD16-), intermediate monocytes (CD14+CD16-), non-classical monocytes (CD14+CD16+) or dendritic cells (CD14-CD16-). Changes in CD169^+^ fraction are indicated as the difference in the %CD169^+^ (Δ%CD169+^+^) measured at the designated time points from baseline pre-infection timepoints (0% = no change).

### Viruses

The barcoded virus model (SIVmac239M) was utilized in this study. SIVmac239M is a virus stock generated from the original Nef-open SIVmac239 clone with a short genetic insertion between the vpx and vpr genes [10,11]. This 34-base genetic insert contains 10 random bases providing over 1 million possible unique sequences representing a viral barcode. The barcode can be sequenced by next generation sequencing thereby generating a genetically diverse virus population that is isogenic outside the viral barcode. In this study, we used a 500IU dose as determined by TZMbl assay which is sufficient to infect all animals with multiple viral lineages without physiologically altering primary replication dynamics [11]. This model represents an intellectual leap forward in how NHP studies can be enhanced to provide both population-based and individual lineage measurements of RCVR establishment, retention during ART, and rebound.

### Barcode sequencing

RNA isolation from plasma was performed using a QIAamp Viral RNA mini kit or QIAgen DSP virus/pathogen Midi kits on the QIASymphony-XP instrument. Complementary DNA (cDNA) was generated with Superscript III reverse transcriptase (Invitrogen) and an SIV-specific reverse primer (Vpr.cDNA3: 5’-CAG GTT GGC CGA TTC TGG AGT GGA TGC-3’). The cDNA was quantified via qRT-PCR using the primers VpxF1 5’-CTA GGG GAA GGA CAT GGG GCA GG-3’ and VprR1 5’-CCA GAA CCT CCA CTA CCC ATT CATC with labeled probe (ACC TCC AGA AAA TGA AGG ACC ACA AAG GG). Prior to sequencing, PCR was performed with VpxF1 and VprR1 primers containing either the F5 or F7 Illumina adaptors with a unique 8-nucleotide index sequence for multiplexing. PCR was performed using High Fidelity Platinum Taq (ThermoFisher). The multiplexed samples were sequenced on a MiSeq instrument (Illumina) and analyzed as previously described [10, 11, 13].

### Viral Detection assays

Plasma SIV RNA and CA-DNA and CA-RNA from cell pellets/tissues were described previously [49, 50]. Intact-proviral DNA assay (IPDA) for SIV was performed as previously described [24].

### Reactivation rate

The viral reactivation rate is defined as the viral growth rate over the average log-ratio of the number of copies of the distinct barcodes detected in rebound plasma [10,13,27,51,52]. While within-group reactivation rates were consistent when several reactivation events were detected (i.e. d7, d9, and d12 animals), this method provides a less reliable estimate in animals that had only 2 rebounding lineages and cannot be applied to animals with only a single rebounding lineage. Because all animals from d3, d5, and d6 animals had only 1 or 2 rebounding viral lineages during treatment interruption, modified reactivation rates were used for inter-group comparisons of RCVR size. The modified viral reactivation rate is defined as the number of distinct barcodes detected in rebound plasma over the “detectable reactivation” time period during which lineages could reactivate and grow to a detectable level in plasma. The beginning of the detectable reactivation window, t_0_, was defined as the expected time for a lineage reactivating immediately upon ART discontinuation to grow to a detectable level, implicitly accounting for drug wash-out and stochasticity of early viral expansion. Here we set t_0_ = 7.1 d, corresponding to the average estimated time that rebounding viral loads of group 9 and 12 animals reached 15 copies/mL, as extrapolated from the viral load curves. The end of the detectable reactivation window t_f_ was defined for each animal as the theoretical last possible time point at which a reactivating lineage could reach the threshold of viral rebound at 15 cp/mL. The theoretical contribution of the last detectable reactivated lineage at the sampled time point was taken as the viral load multiplied by the reciprocal sequencing input, and t_f_ was back calculated based on the piecewise viral load curve.

### Statistics

Statistical analysis was performed using R, survival analysis using Prism, and model fitting using Monolix. Kaplan-Meier survival curves for time to detection after ART discontinuation were estimated for each ART initiation group. The logrank test was used to assess if survival (defined as no measurable viral rebound) differed across groups, and the logrank test for trend was used to assess if there was a linear trend between the day of ART initiation and the median survival time. Finally, the logrank test was used to assess if each pair of groups differed significantly in survival, with Benjamini-Hochberg adjustment of p-values to account for multiple comparisons. The Kruskall-Wallis Test was used to assess if the reactivation rate and the log_10_-transformed per-virion reactivation rate differed across ART initiation groups. Wilcoxon-tests were used to look for significant differences in these measures between each pair of groups, with Benjamini-Hochberg adjustment of p-values. Pearson correlation analysis was used to assess the strength of association between the values of different virological measures and estimated reactivation rates. To determine how RR depends on pre-ART peak viral load, linear and logistic models were fit to the data via maximum likelihood in Monolix. Animals that did not rebound were treated as left-censored and could take any values below the minimum detectable reactivation rate of 1 over last day of follow-up. Model fits were compared based on the Akaike information criteria.

## Supporting information

S1 Fig The mean (+ s.e.m.) change of CD169 expression levels on CD14^+^ monocytes in blood of rhesus macaques stratified by time of cART initiation. Animals from all groups with ART initiation at day 5 or later show an increase in monocyte activation as evidenced by induction CD169 expression starting at day 6 post SIVmac239M infection, a response that is largely abrogated with ART initiation at day 3 or 4. The maximal responses in the day 9 and 12 ART-initiation groups plateau at day 9 post-infection and are fully resolved by day 21 post-infection.
(PDF)

S2 Fig Early pre- and post-ART plasma viral load dynamics of individual RMs. Plasma viral loads of individual RMs within each day of ART initiation group is shown. ART initiation is shown by colored dashed line. The average peak, range and AUC are listed for each group. Groups are color-coded based on days post infection until ART: d3 (blue), d4 (red), d5 (green), d6 (purple), d7 (orange), d9 (black) and d12 (brown).
(PDF)

S3 Fig Barcode distribution and plasma viral load dynamics of 3 noncontrolling RMs. In three of 35 RMs (A5, B5, D5) with ART starting at day 3, 4, and 6, pVL remained above 15 copies/ml for 140 days, 380 days, or through the end of the study. Barcodes were assessed at peak pVL (left panel) for each RM. In addition, 5 to 15 additional time points were used to measure barcode distribution on ART. In all cases, only a single barcode was detected in each RM during these on ART plasma sequences and that individual barcode is the only one detectable during off-ART rebound. Additional pol sequencing revealed wild-type virus in Int/RT (*) in all cases except in RM B5 where fixed mutations (^) in RT: K65R, A62V, V202A, S205L and INT: E92Q, D163G were detected at day 350 while on ART which was also retained during off-ART rebound.
(PDF)

S4 Fig Early CA-DNA accumulation prior to and following ART initiation. CA-DNA was measured for individual RMs within each ART initiation group. ART initiation is shown by dashed line.
(PDF)

S5 Fig Early CA-RNA accumulation prior to and following ART initiation. CA-RNA was measured for individual RMs within each ART initiation group. ART initiation is shown by dashed line.
(PDF)

S6 Fig Delay in first phase viral decay kinetics when ART is started pre-peak. The time from ART initiation until exponential decay is shown. The number of days between ART and decay are shown by vertical line with the days listed for each RM. Groups are color-coded based on days post infection until ART: d3 (blue), d4 (red), d5 (green), d6 (purple), d7 (orange), d9 (black) and d12 (brown).
(PDF)

S7 Fig Delay in first phase viral decay kinetics when ART is started pre-peak. The time from ART initiation until onset of exponential decay is shown from 63 animals previously published but also infected with SIVmac239 and initiating ART between 4 and 42 dpi [18]. The number of days between ART and decay are shown by vertical line with the days listed for each RM. Groups are color-coded based on days post infection until ART: 4–5 (red), 6 (purple), 7 (orange), 8–9 (black), 12 (brown), and 42 (pink).
(PDF)

S8 Fig Total pVL measurements from challenge to treatment interruption. PVL of individual RMs within each ART initiation group are shown. Based on shift to higher sensitivity assay once viremia was suppressed, assay threshold changed from 15 copies/mL to 1 copy/mL. Open symbols represent samples with no viral signal detected (<1 or <15 copies/mL).
(PDF)

S9 Fig CA-RNA and CA-DNA viral load measurements from PBMC, peripheral LNMCs, DUO and BM from 3, 70, and 250 dpi. Unfilled symbols represent samples with no viral signal detected and are plotted at the calculated limit of detection (LOD) for each sample based on specimen input. Groups are color-coded based on days post infection until ART: d3 (blue), d4 (red), d5 (green), d6 (purple), d7 (orange), d9 (black) and d12 (brown).
(PDF)

S10 Fig CD8 depletion fails to cause rebound in non-rebounding off-ART RMs. CD8α^+^ cell depletion was performed three times in one month in all 7 RMs that failed to rebound off-ART naturally. No measurable plasma viremia was detected.
(PDF)

S11 Fig Pre-ART plasma barcode proportion during primary infection. Samples from all RMs with peak pVL greater than 380 copies/ml were subjected to barcode sequencing. While the number of detectable barcodes per RM increased as pVL increased, in part due to increases in limit of detection based on input (black line), the average number of detectable barcodes pre-ART was 67 (range 11–135; n = 28). Groups are color-coded based on days post infection until ART: d3 (blue), d4 (red), d5 (green), d6 (purple), d7 (orange), d9 (black) and d12 (brown).
(PDF)

S12 Fig Consistent barcode distribution across multiple tissues during primary infection. Plasma viral RNA barcode distribution was compared with barcodes in up to 5 distinct sites including lymph node mononuclear cells (LNMCs), bone marrow (BM), peripheral blood mononuclear cells (PBMCs), duodenum (DUO) and bronchial alveolar lavage (BAL). Groups are color-coded based on days post infection until ART: d5 (green), d6 (purple), d7 (orange), d9 (black) and d12 (brown).
(PDF)

S13 Fig No viral measurement predicts off-ART rebound or not in d3 or d4 ART RMs. The stochastic nature of RCVR generation and maintenance is highlighted in RM A4 (dark blue), which did not show any increased viral measurement in either pre-ART or during ART compared to other d3 (light blue) or d4 (red) RMs. Decay kinetics of CA-RNA and CA-RNA within PBMC (circle), LNMC (square), DUO (up triangle) and BM (down triangle) from 3-, 70- or 250-days following ART initiation.
(PDF)

S14 Fig Barcode comparisons pre-ART versus post-ART rebound. Rebound kinetics and barcodes for each rebounding RM based on day ART initiated (left panel). Barcode comparisons across time post rebound and compared to the pre-ART population (right panel). Groups are color-coded based on days post infection until ART: d6 (purple), d7 (orange), d9 (black) and d12 (brown).
(PDF)

S15 Fig Viral measurements do not predict off-ART rebound in d4 and d5 ART RMs. The stochastic nature of RCVR generation and maintenance is highlighted in RMs B1 and B2 (dark red), which failed to rebound despite viral measurements slightly lower than the comparable d5 RMs (green). Decay kinetics of CA-RNA and CA-RNA within PBMC (circle), LNMC (square), DUO (up triangle) and BM (down triangle) from 3-, 70- or 250-days following ART initiation.
(PDF)

S1 Data Excel spreadsheet containing, in separate sheets, the underlying numerical data and statistical analysis for Figs 1–7 and all supplemental figures. (XLSX)
