# Correction: Early antiretroviral therapy in SIV-infected rhesus macaques reveals a multiphasic, saturable dynamic accumulation of the rebound competent viral reservoir

**DOI:** 10.1371/journal.ppat.1014399

**Published:** 2026-07-06

**Authors:** The PLOS Pathogens Staff

## Notice of Republication

This article was republished on June 2 2026, to correct errors in the XML version of the paper where the Supporting Information images were not showing.
