# Supplementary figures and images for: Early antiretroviral therapy in SIV-infected rhesus macaques reveals a multiphasic, saturable dynamic accumulation of the rebound competent viral reservoir
Source: PLoS Pathog. 2024 Apr 9;20(4):e1012135. doi: 10.1371/journal.ppat.1012135 (PMC11003637; doi:10.1371/journal.ppat.1012135)

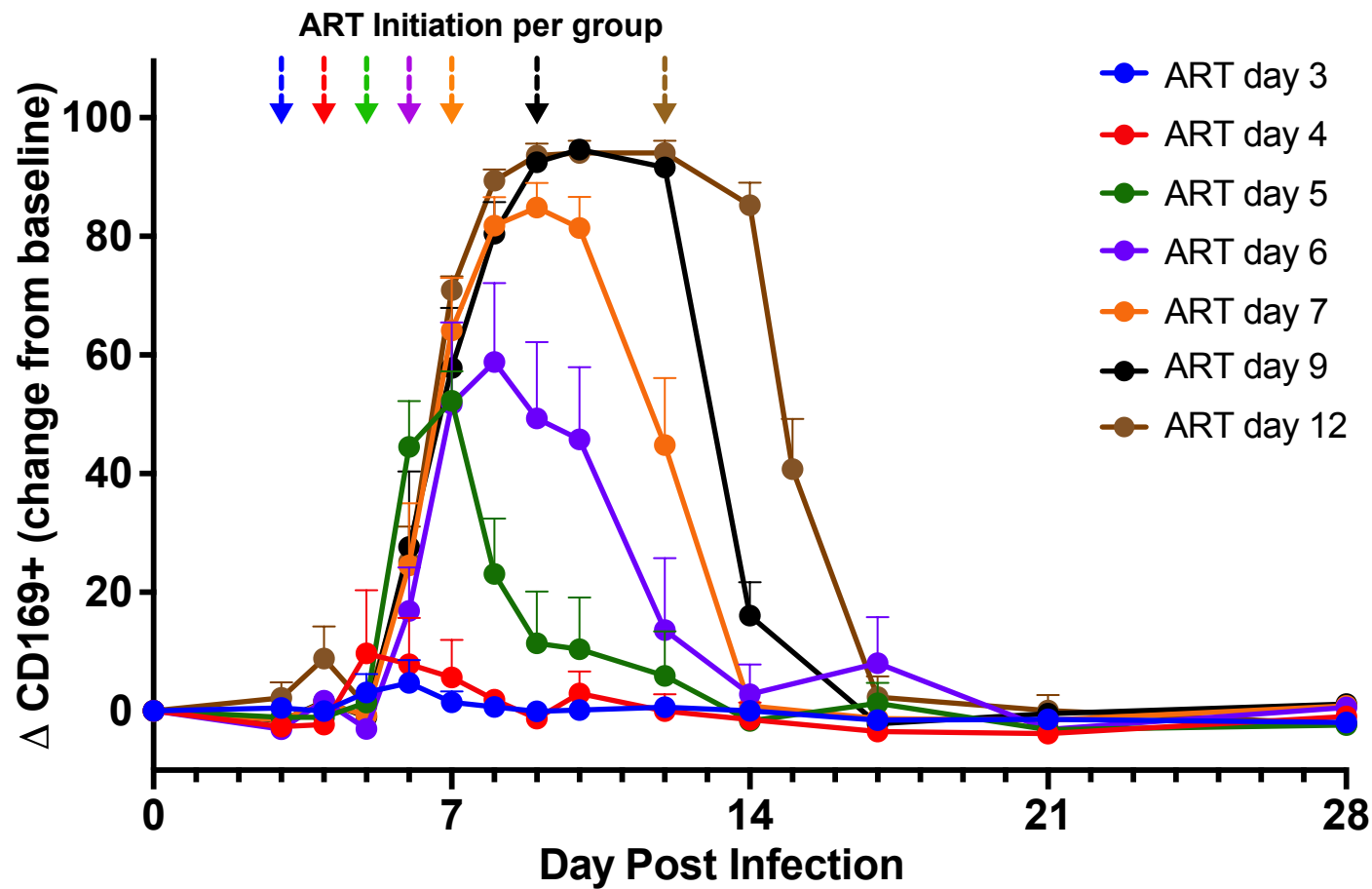

Supplement: S1 Fig — Animals from all groups with ART initiation at day 5 or later show an increase in monocyte activation as evidenced by induction CD169 expression starting at day 6 post SIVmac239M infection, a response that is largely abrogated with ART initiation at day 3 or 4. The maximal responses in the day 9 and 12 ART-initiation groups plateau at day 9 post-infection and are fully resolved by day 21 post-infection. (PDF) [file ppat.1012135.s001.pdf]

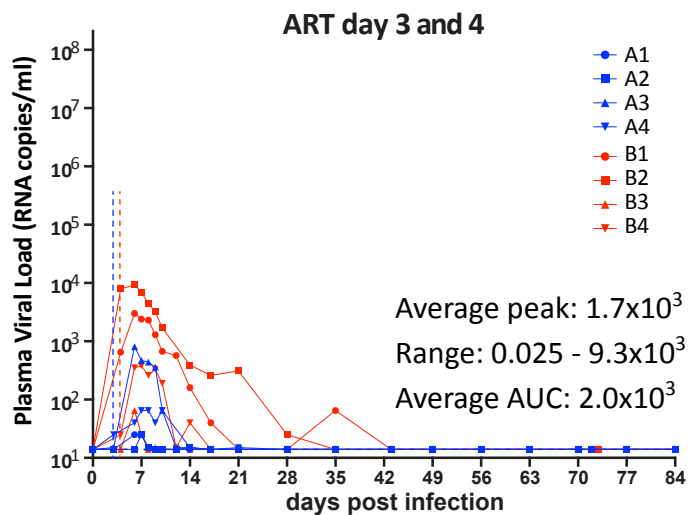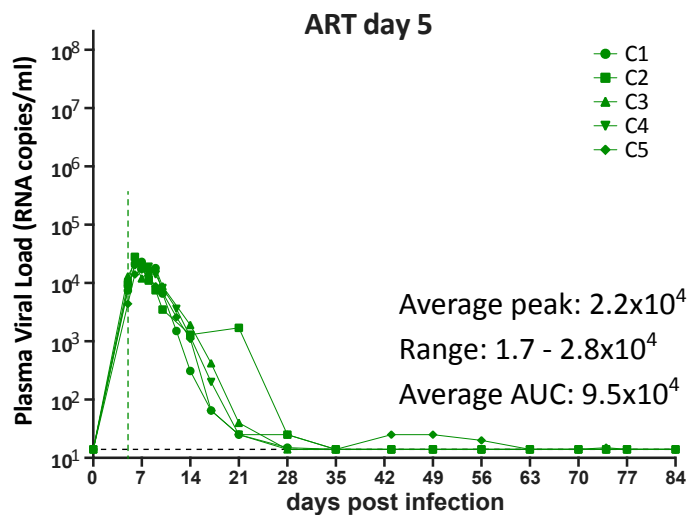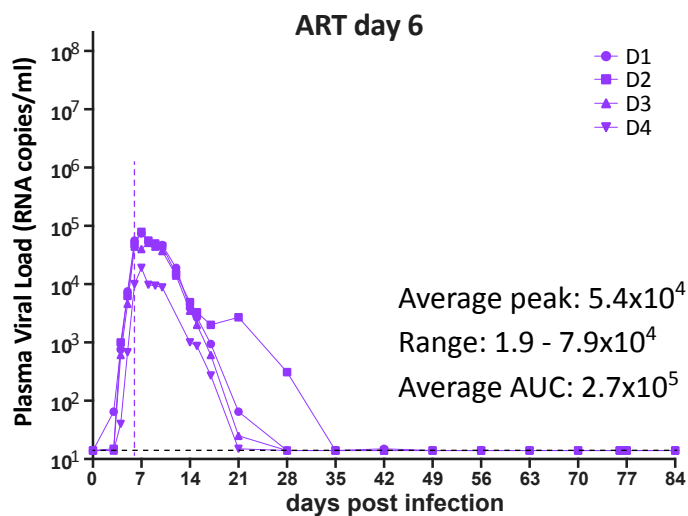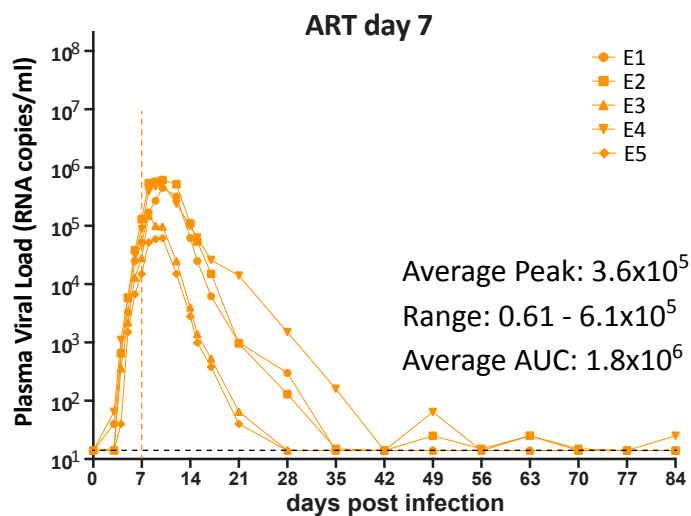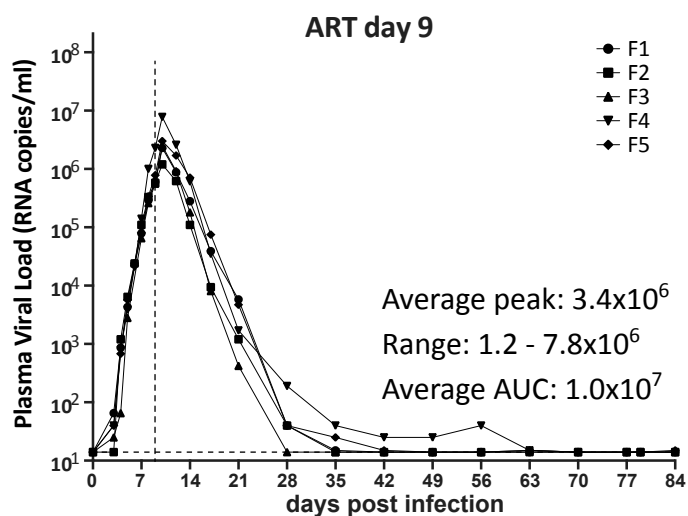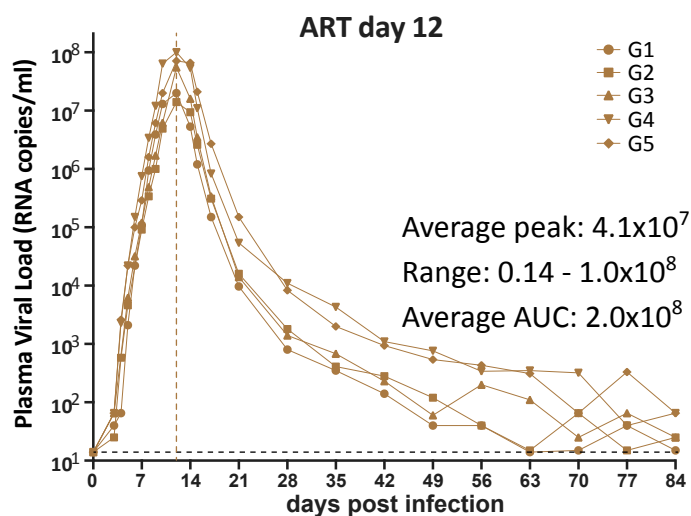

Supplement: S2 Fig — Plasma viral loads of individual RMs within each day of ART initiation group is shown. ART initiation is shown by colored dashed line. The average peak, range and AUC are listed for each group. Groups are color-coded based on days post infection until ART: d3 (blue), d4 (red), d5 (green), d6 (purple), d7 (orange), d9 (black) and d12 (brown). (PDF) [file ppat.1012135.s002.pdf]

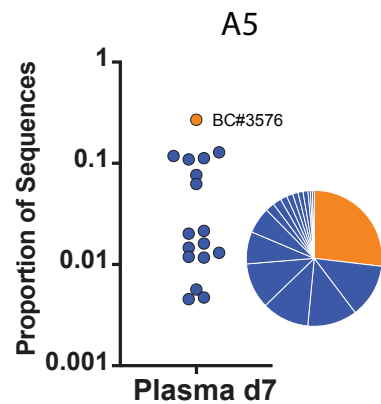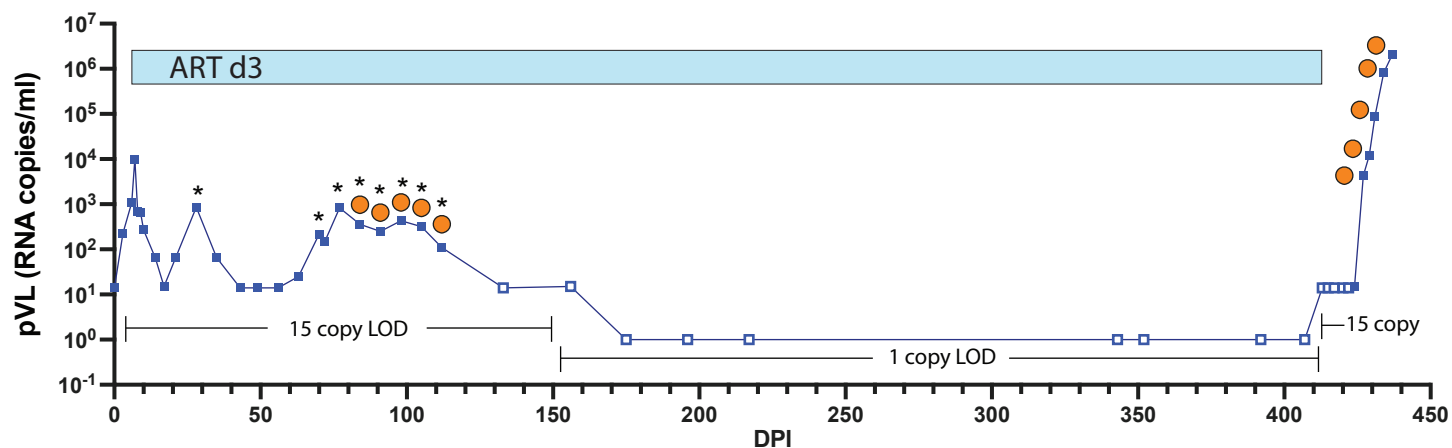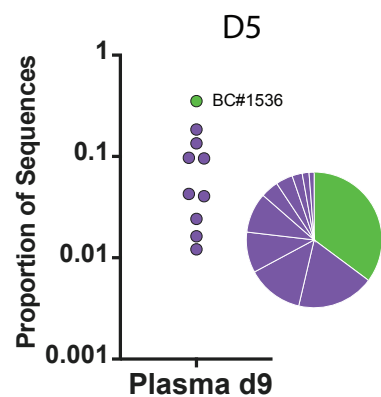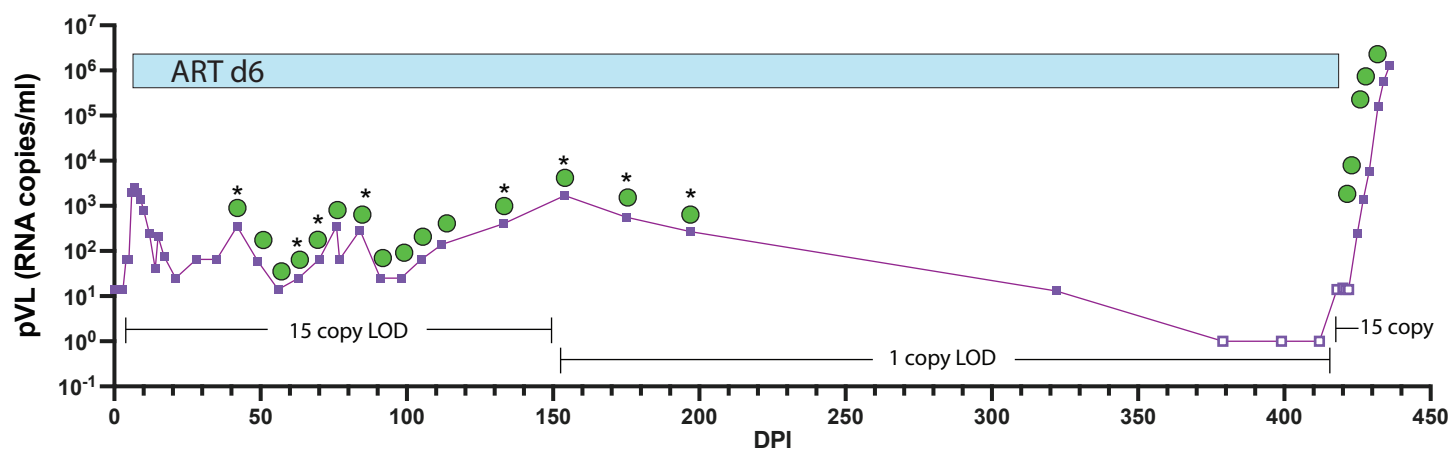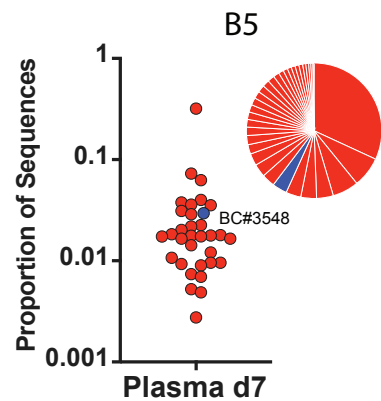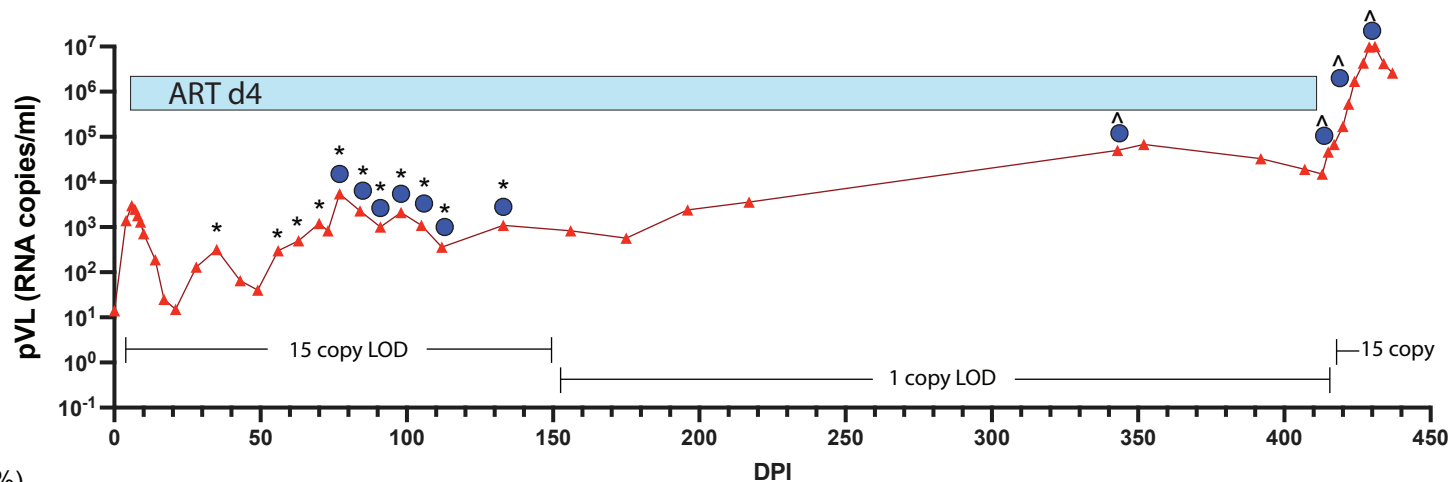

\* WT INT/RT (>99.9%)

^ Fixed POL Mutations: RT: K65R, A62V, V202A, S205L. INT: E92Q, D163G

Supplement: S3 Fig — In three of 35 RMs (A5, B5, D5) with ART starting at day 3, 4, and 6, pVL remained above 15 copies/ml for 140 days, 380 days, or through the end of the study. Barcodes were assessed at peak pVL (left panel) for each RM. In addition, 5 to 15 additional time points were used to measure barcode distribution on ART. In all cases, only a single barcode was detected in each RM during these on ART plasma sequences and that individual barcode is the only one detectable during off-ART rebound. Additional pol sequencing revealed wild-type virus in Int/RT (*) in all cases except in RM B5 where fixed mutations (^) in RT: K65R, A62V, V202A, S205L and INT: E92Q, D163G were detected at day 350 while on ART which was also retained during off-ART rebound. (PDF) [file ppat.1012135.s003.pdf]

Day 3 ART

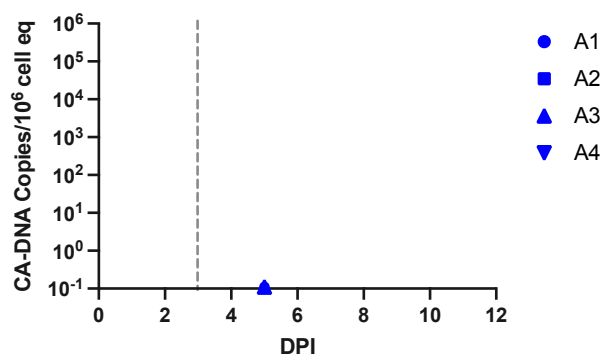

Day 4 ART

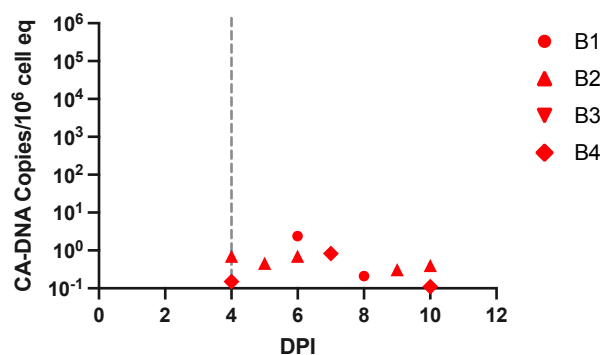

Day 5 ART

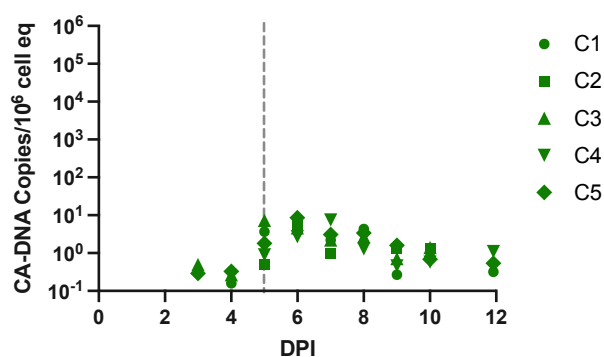

Day 6 ART

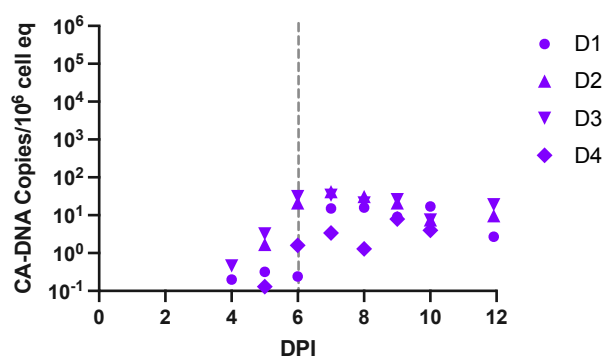

Day 7 ART

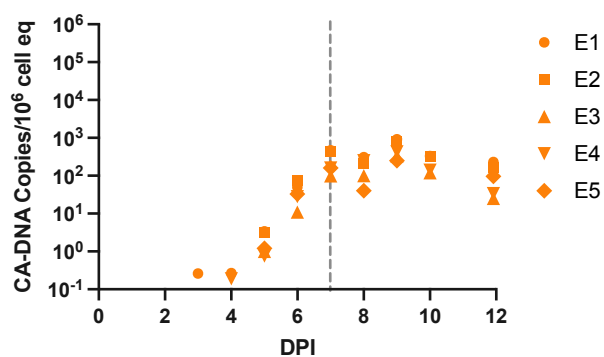

Day 9 ART

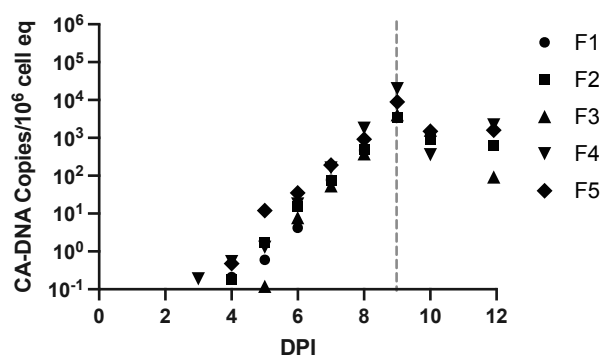

Day 12 ART

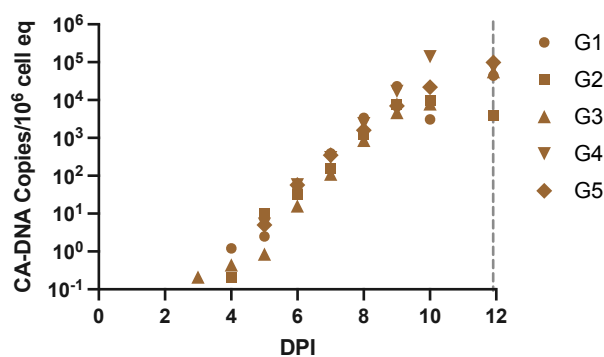

Supplement: S4 Fig — CA-DNA was measured for individual RMs within each ART initiation group. ART initiation is shown by dashed line. (PDF) [file ppat.1012135.s004.pdf]

Day 3 ART

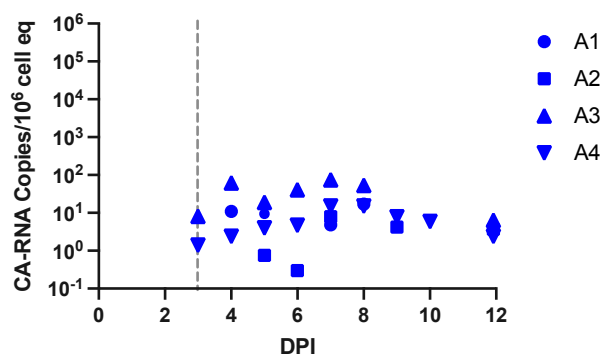

Day 4 ART

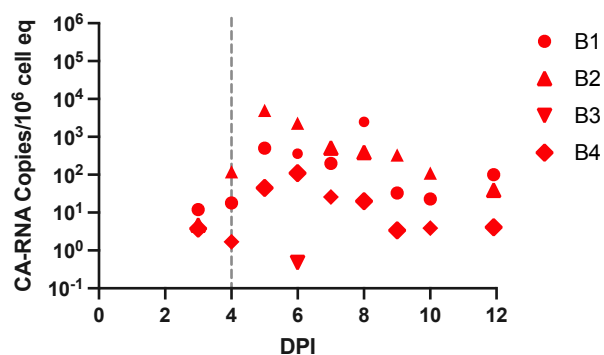

Day 5 ART

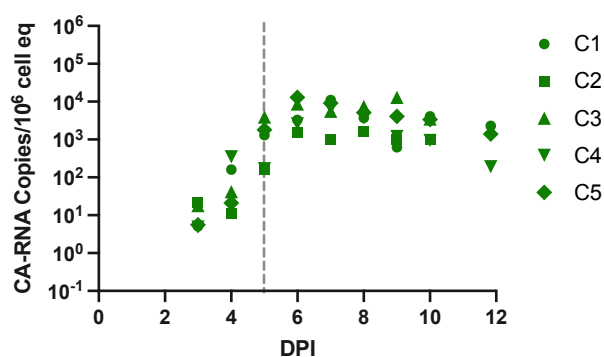

Day 6 ART

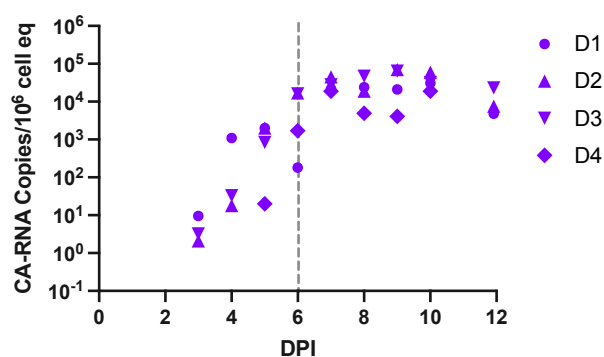

Day 7 ART

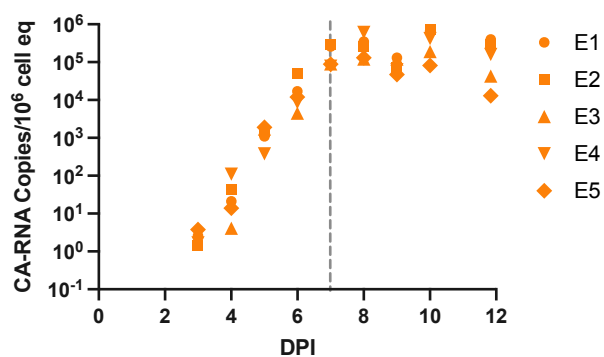

Day 9 ART

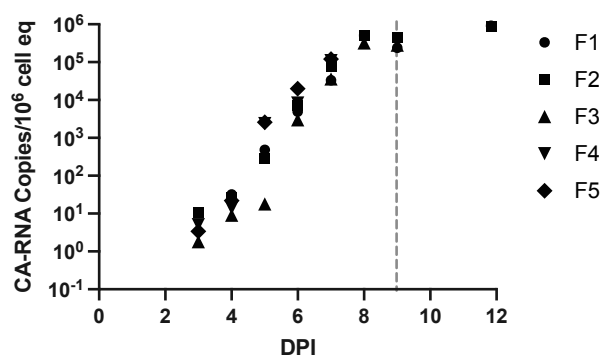

Day 12 ART

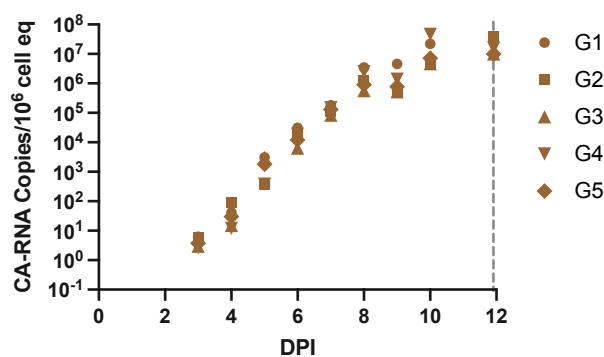

Supplement: S5 Fig — CA-RNA was measured for individual RMs within each ART initiation group. ART initiation is shown by dashed line. (PDF) [file ppat.1012135.s005.pdf]

Plasma vRNA copies (log10)

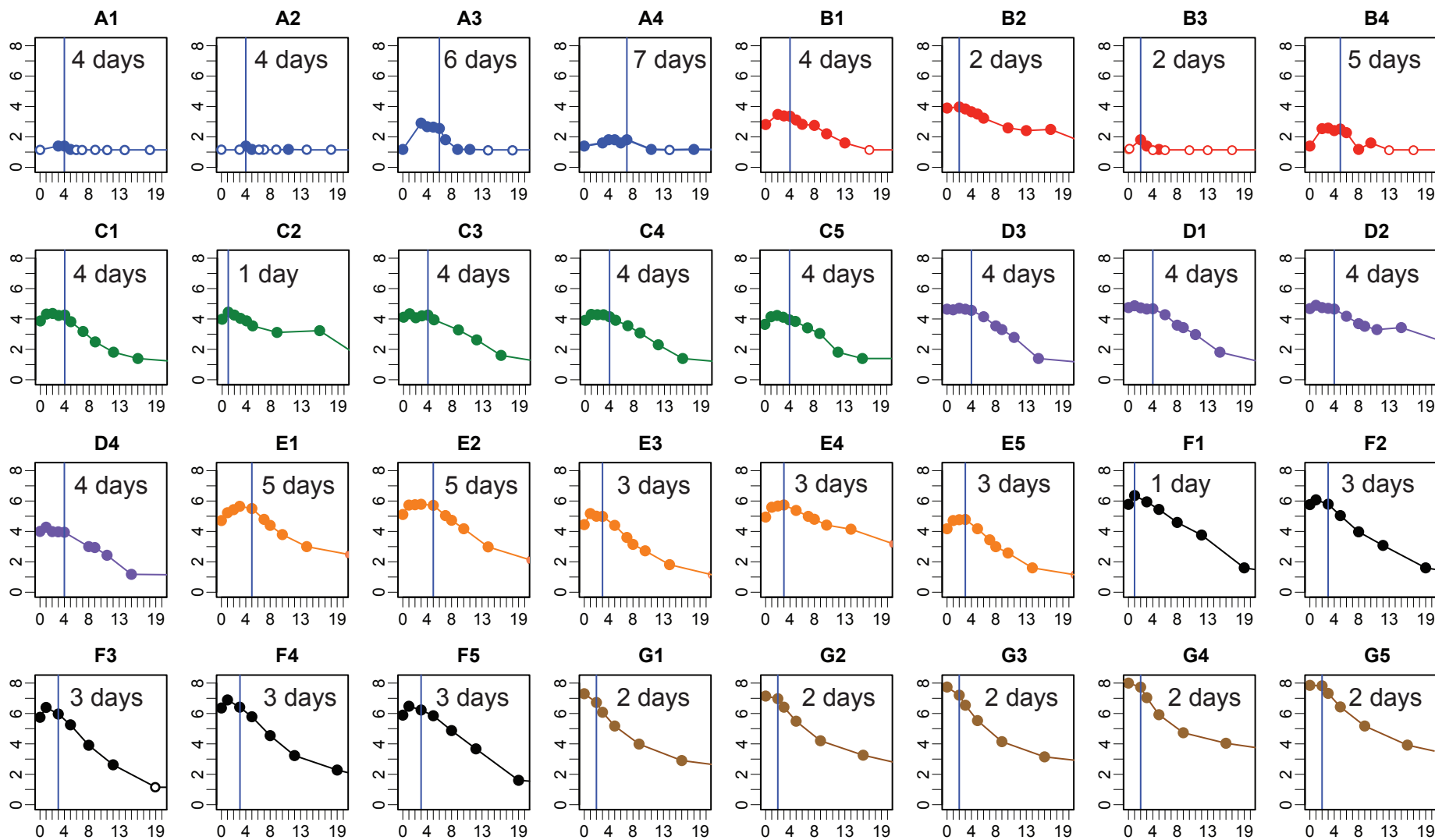

Days post ART initiation

Supplement: S6 Fig — The time from ART initiation until exponential decay is shown. The number of days between ART and decay are shown by vertical line with the days listed for each RM. Groups are color-coded based on days post infection until ART: d3 (blue), d4 (red), d5 (green), d6 (purple), d7 (orange), d9 (black) and d12 (brown). (PDF) [file ppat.1012135.s006.pdf]

Plasma vRNA copies (log10)

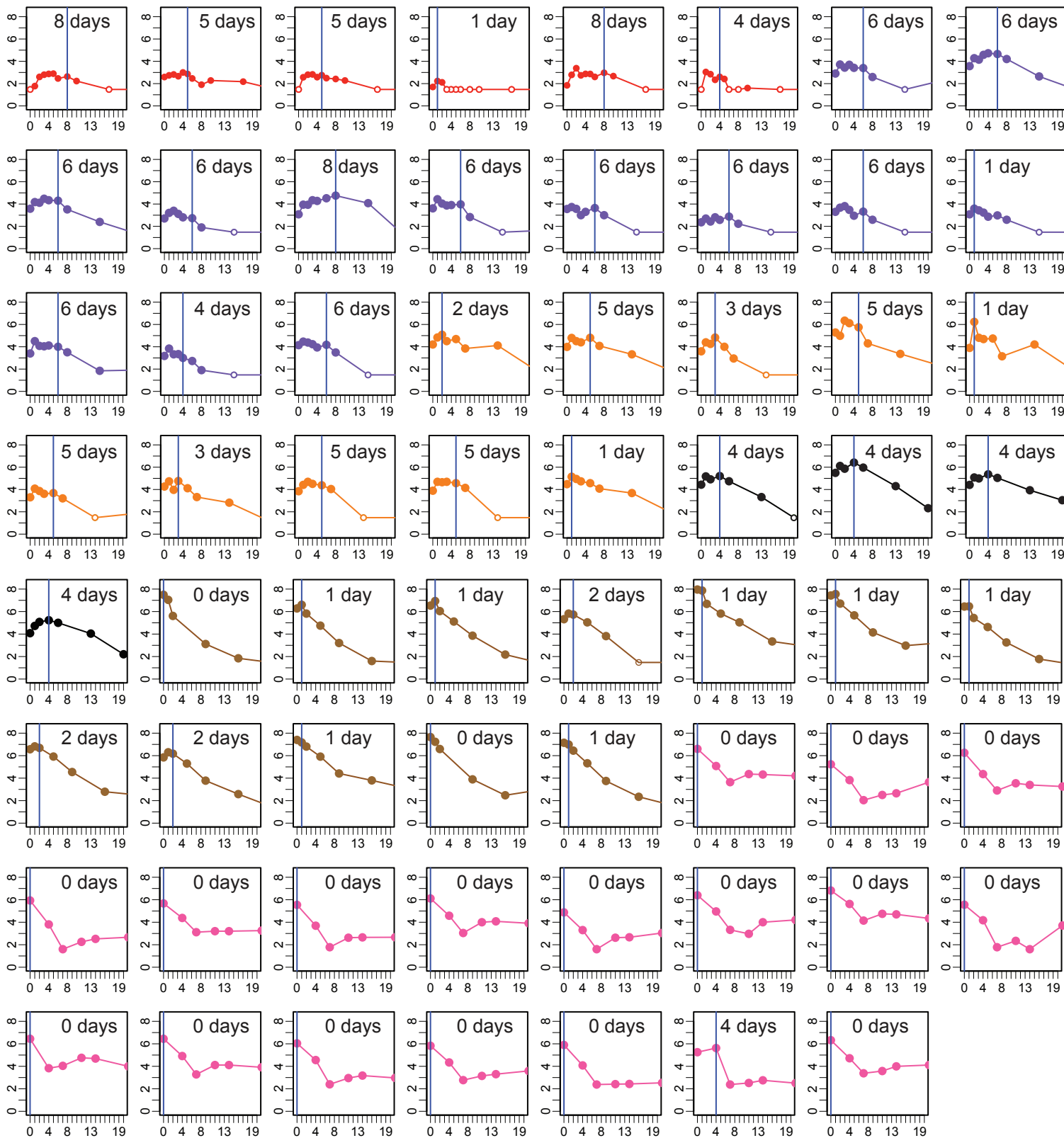

Days post ART initiation

Supplement: S7 Fig — The time from ART initiation until onset of exponential decay is shown from 63 animals previously published but also infected with SIVmac239 and initiating ART between 4 and 42 dpi [18]. The number of days between ART and decay are shown by vertical line with the days listed for each RM. Groups are color-coded based on days post infection until ART: 4–5 (red), 6 (purple), 7 (orange), 8–9 (black), 12 (brown), and 42 (pink). (PDF) [file ppat.1012135.s007.pdf]

**Days 3 and 4**

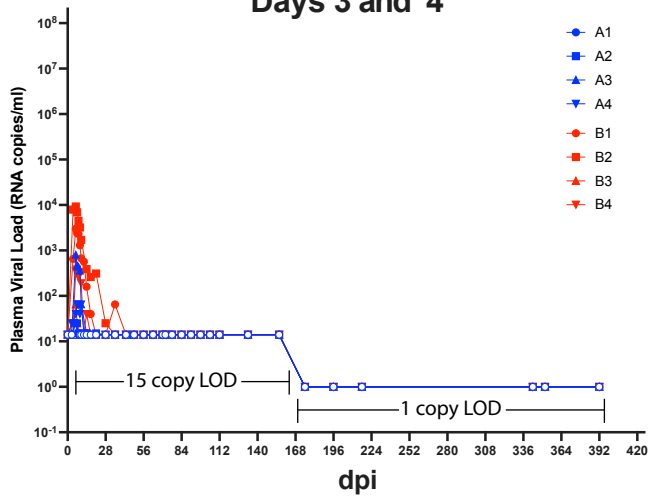

**Day 5**

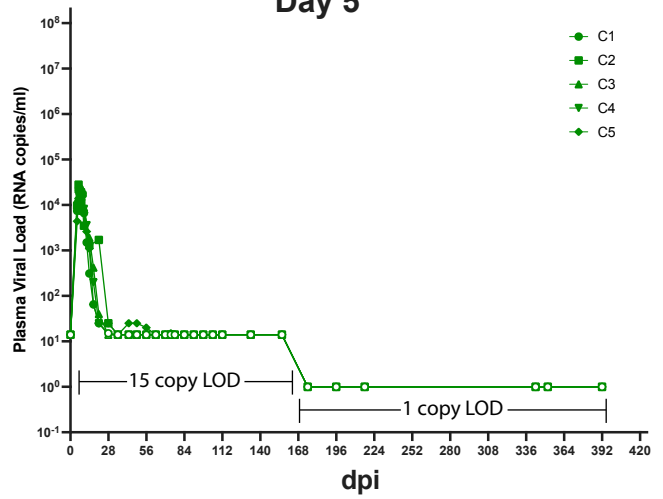

**Day 6**

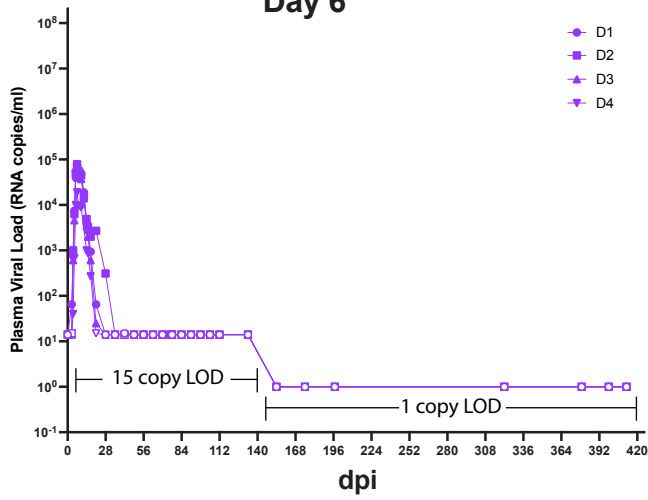

**Day 7**

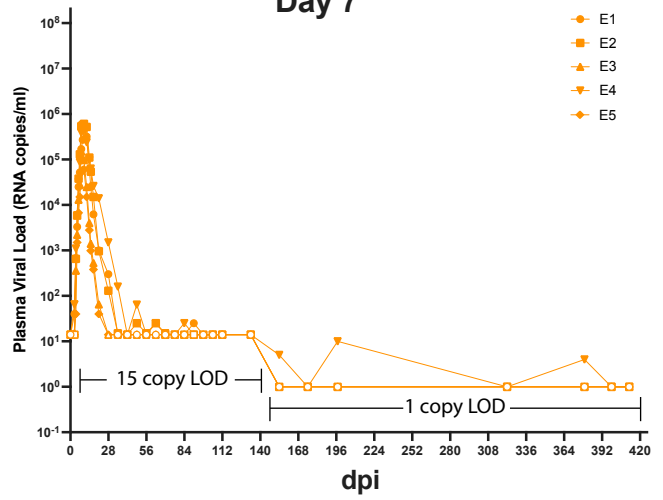

**Day 9**

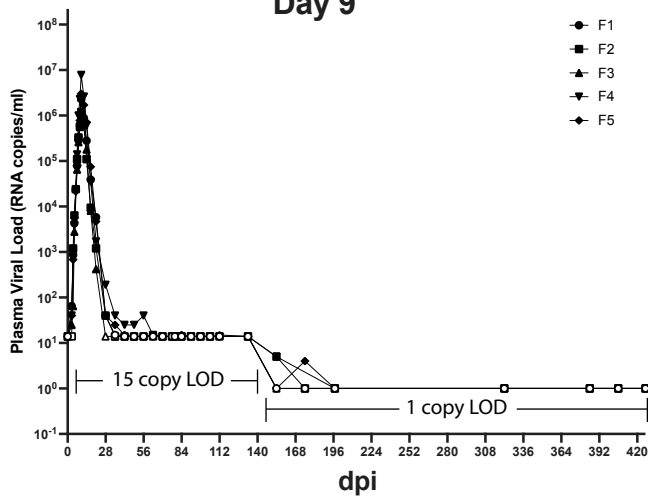

**Day 12**

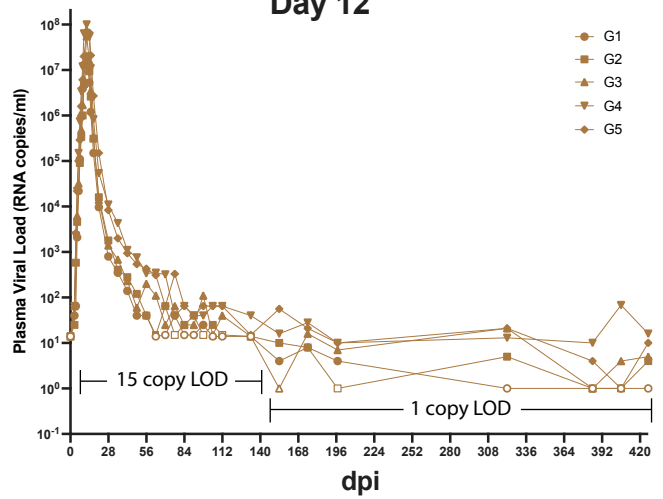

Supplement: S8 Fig — PVL of individual RMs within each ART initiation group are shown. Based on shift to higher sensitivity assay once viremia was suppressed, assay threshold changed from 15 copies/mL to 1 copy/mL. Open symbols represent samples with no viral signal detected (<1 or <15 copies/mL). (PDF) [file ppat.1012135.s008.pdf]

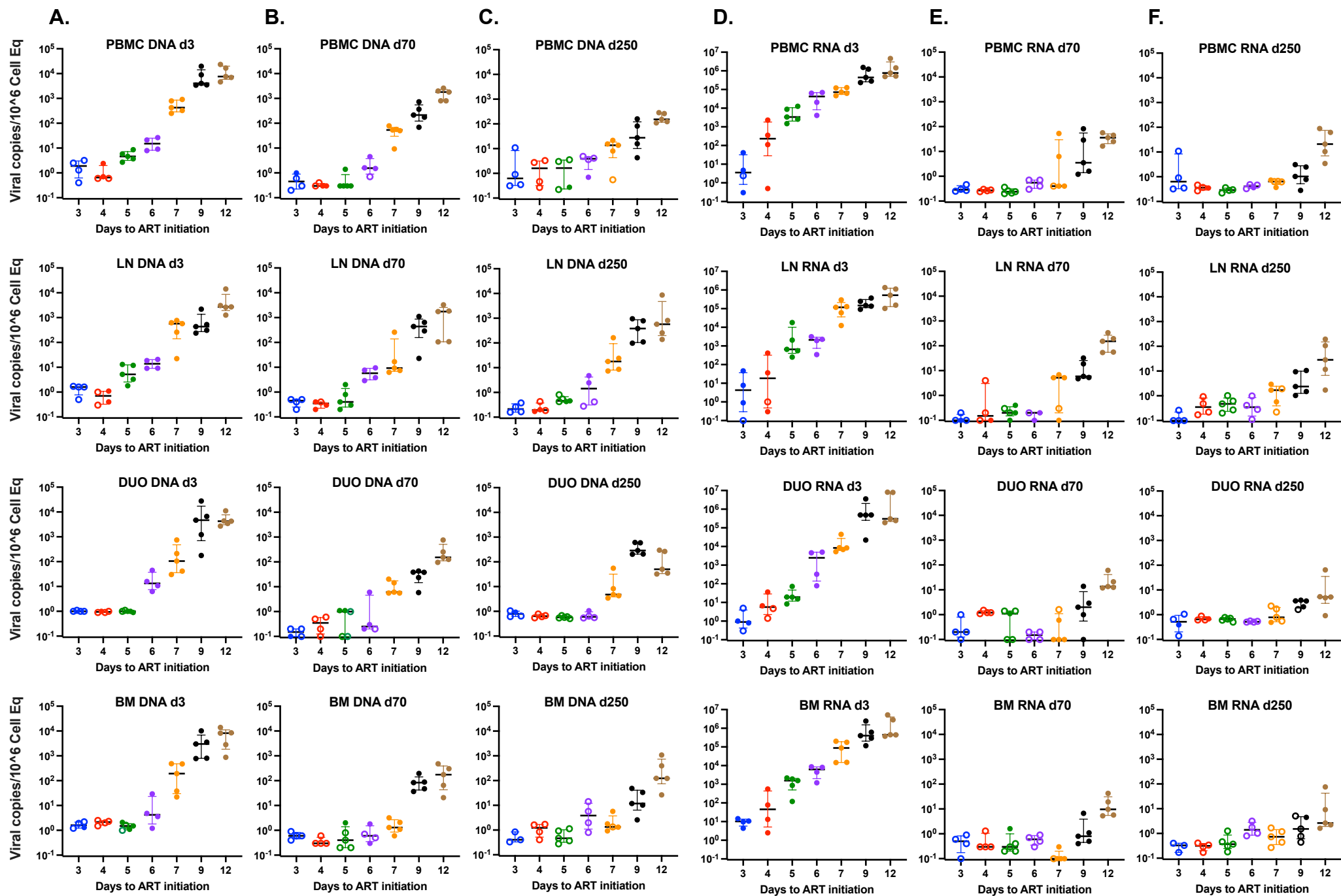

Supplement: S9 Fig — Unfilled symbols represent samples with no viral signal detected and are plotted at the calculated limit of detection (LOD) for each sample based on specimen input. Groups are color-coded based on days post infection until ART: d3 (blue), d4 (red), d5 (green), d6 (purple), d7 (orange), d9 (black) and d12 (brown). (PDF) [file ppat.1012135.s009.pdf]

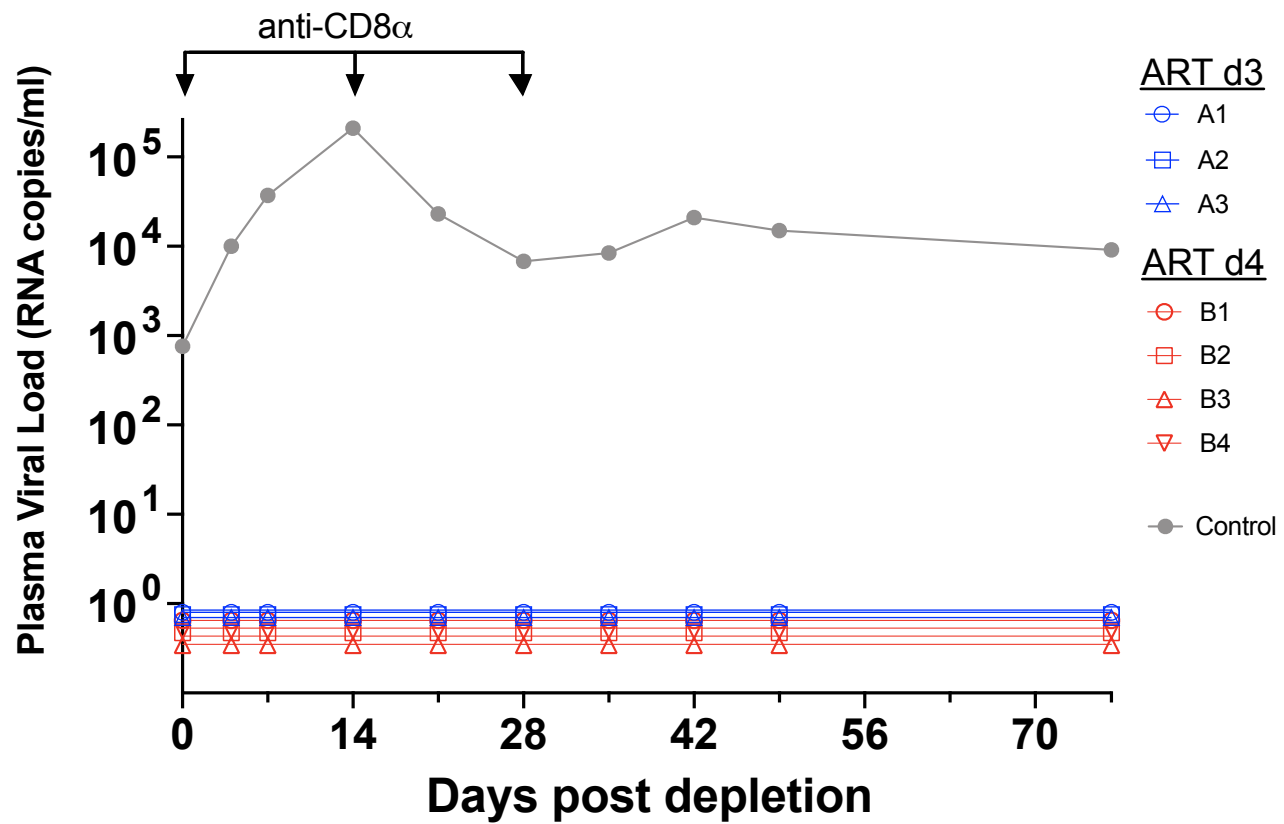

Supplement: S10 Fig — CD8α+ cell depletion was performed three times in one month in all 7 RMs that failed to rebound off-ART naturally. No measurable plasma viremia was detected. (PDF) [file ppat.1012135.s010.pdf]

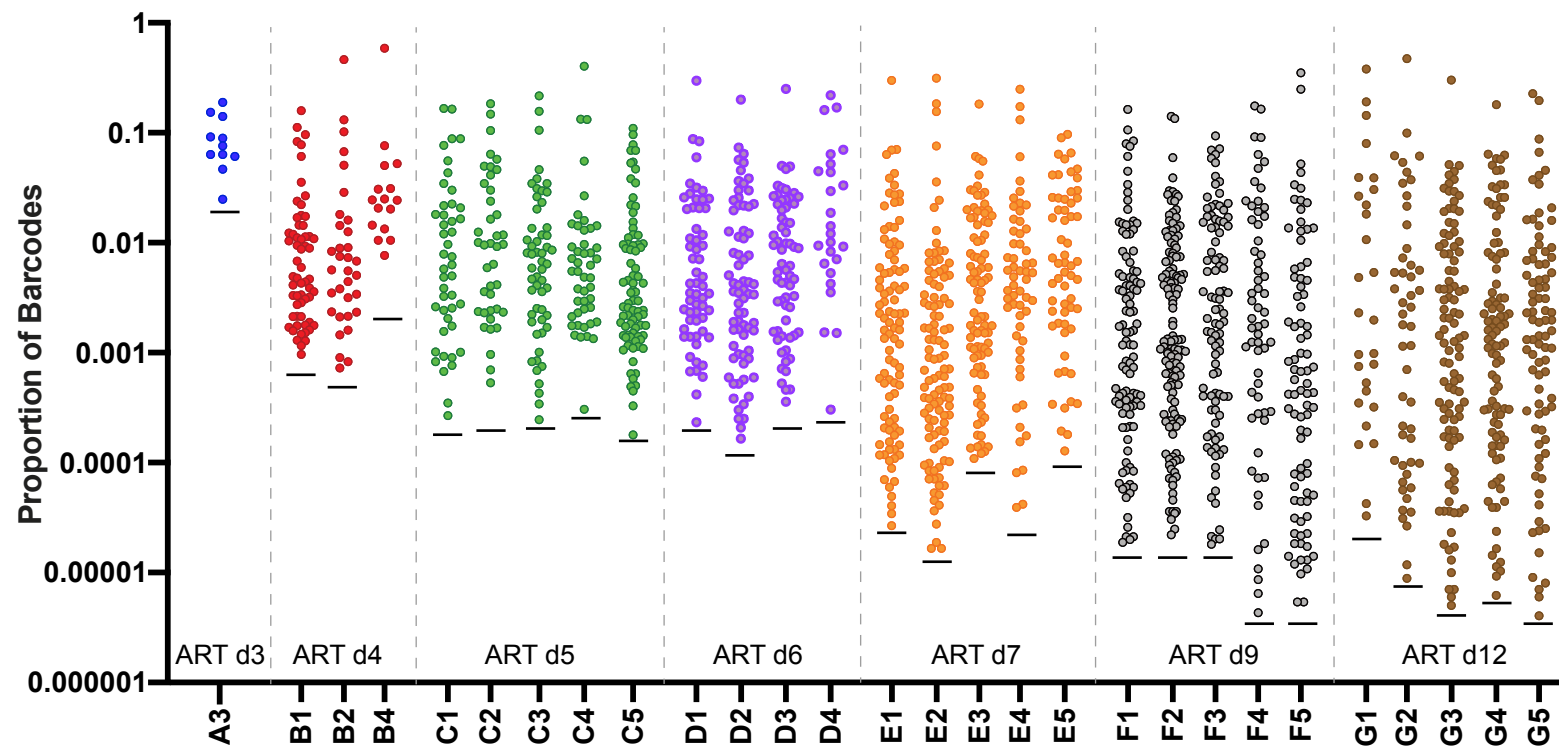

Supplement: S11 Fig — Samples from all RMs with peak pVL greater than 380 copies/ml were subjected to barcode sequencing. While the number of detectable barcodes per RM increased as pVL increased, in part due to increases in limit of detection based on input (black line), the average number of detectable barcodes pre-ART was 67 (range 11–135; n = 28). Groups are color-coded based on days post infection until ART: d3 (blue), d4 (red), d5 (green), d6 (purple), d7 (orange), d9 (black) and d12 (brown). (PDF) [file ppat.1012135.s011.pdf]

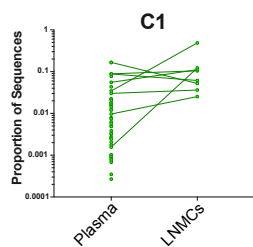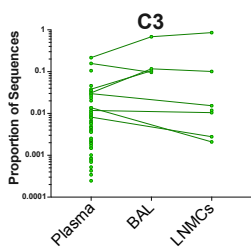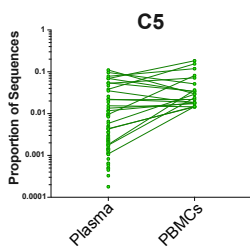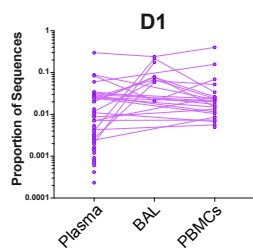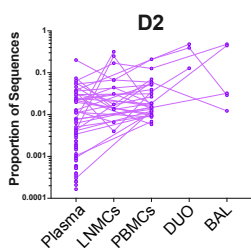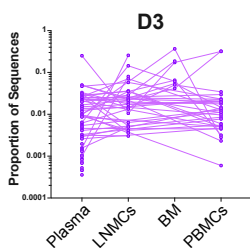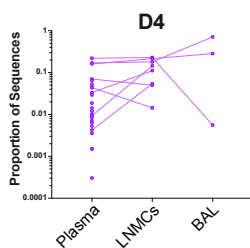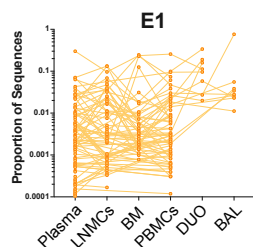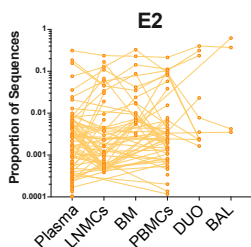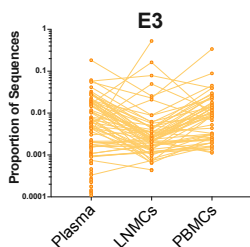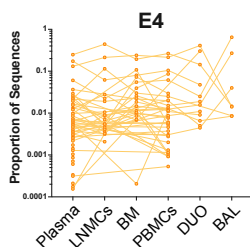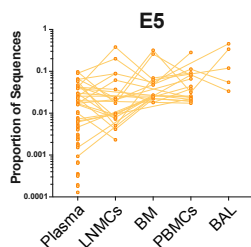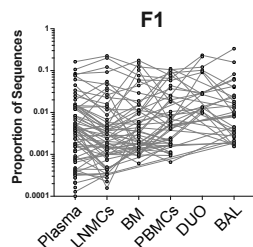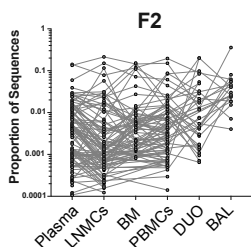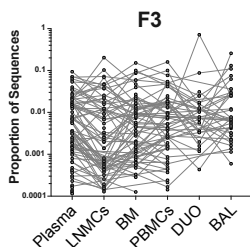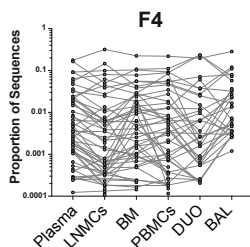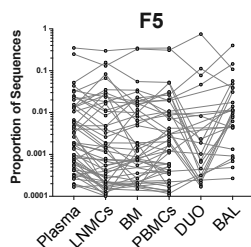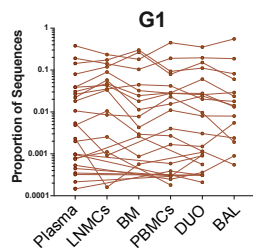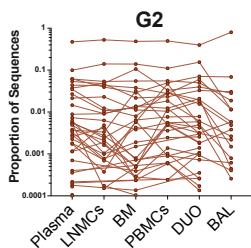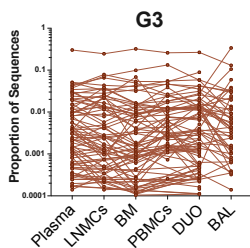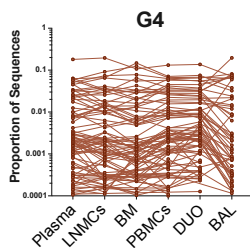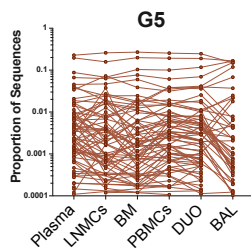

Supplement: S12 Fig — Plasma viral RNA barcode distribution was compared with barcodes in up to 5 distinct sites including lymph node mononuclear cells (LNMCs), bone marrow (BM), peripheral blood mononuclear cells (PBMCs), duodenum (DUO) and bronchial alveolar lavage (BAL). Groups are color-coded based on days post infection until ART: d5 (green), d6 (purple), d7 (orange), d9 (black) and d12 (brown). (PDF) [file ppat.1012135.s012.pdf]

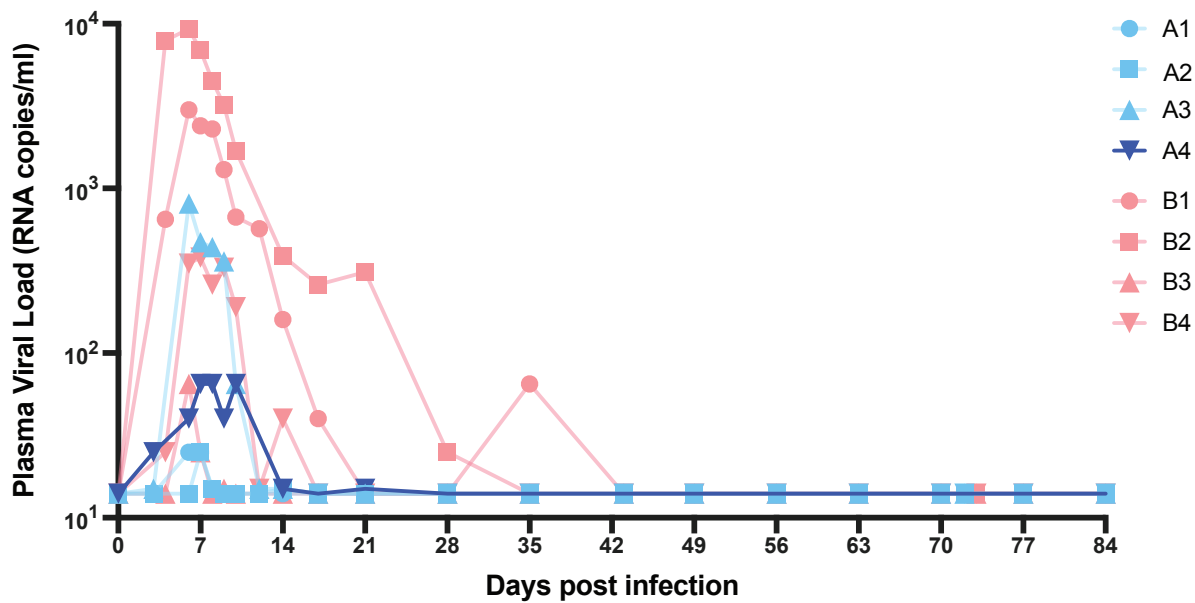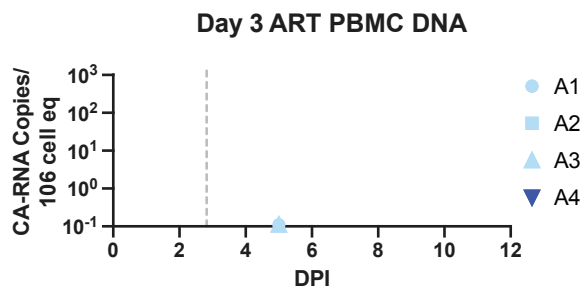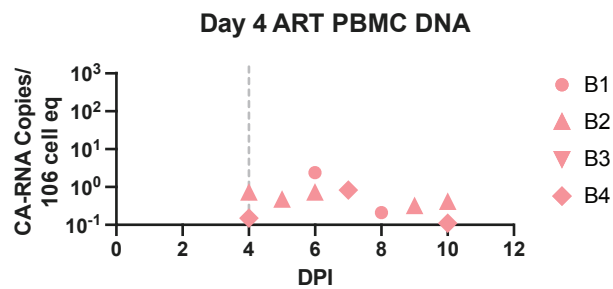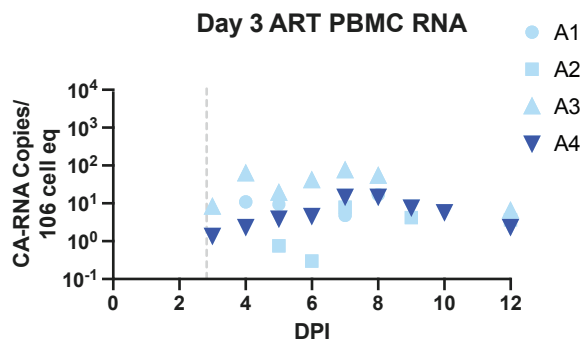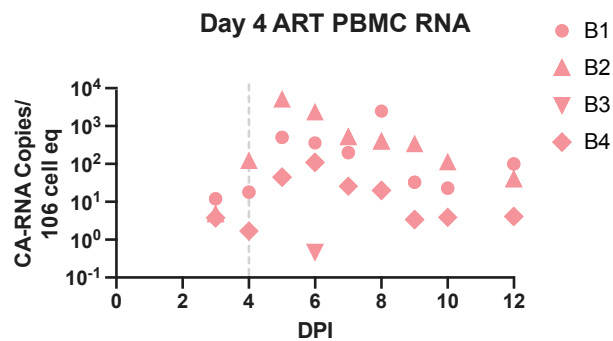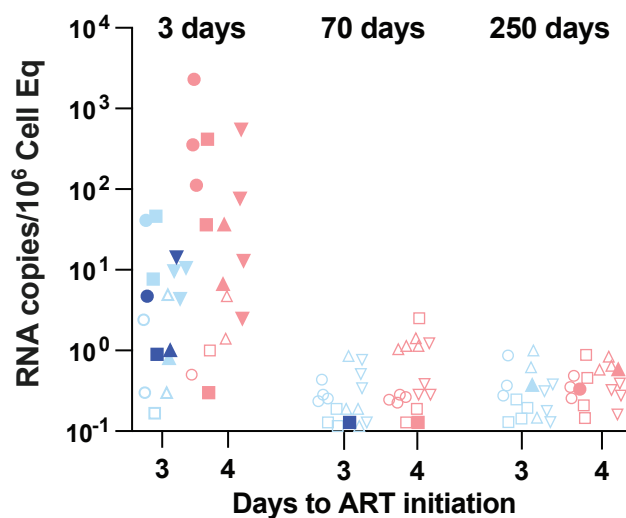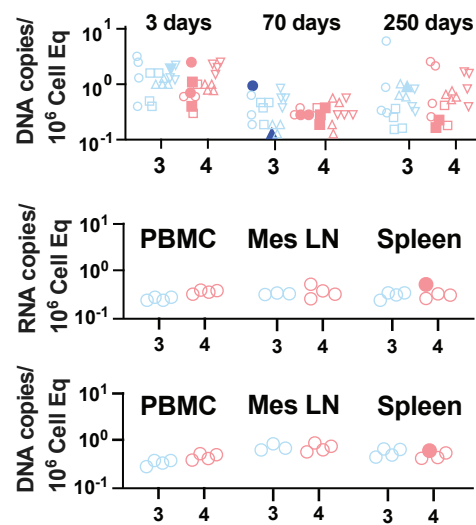

Supplement: S13 Fig — The stochastic nature of RCVR generation and maintenance is highlighted in RM A4 (dark blue), which did not show any increased viral measurement in either pre-ART or during ART compared to other d3 (light blue) or d4 (red) RMs. Decay kinetics of CA-RNA and CA-RNA within PBMC (circle), LNMC (square), DUO (up triangle) and BM (down triangle) from 3-, 70- or 250-days following ART initiation. (PDF) [file ppat.1012135.s013.pdf]

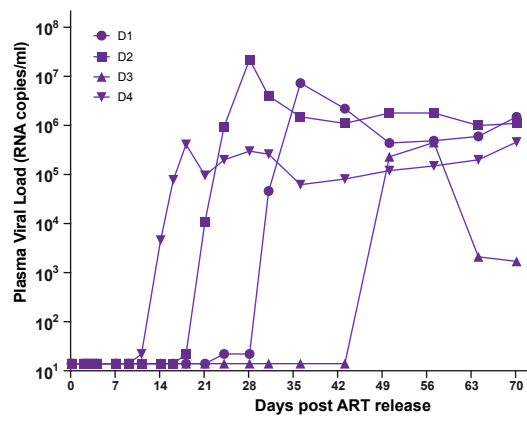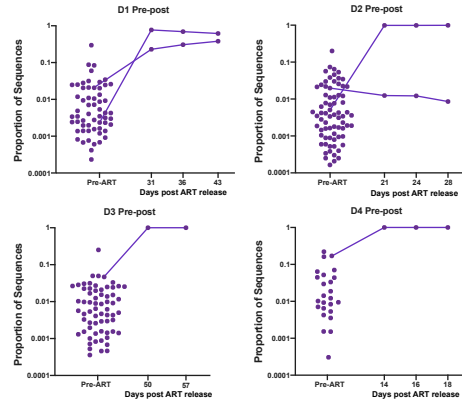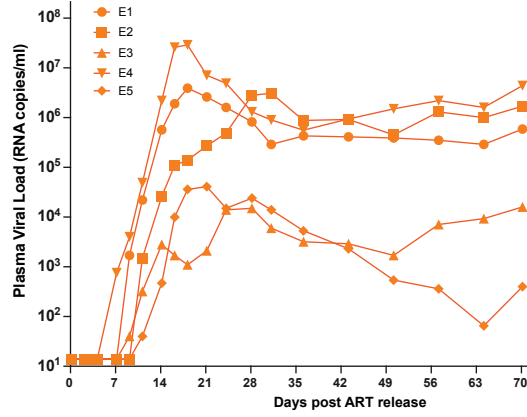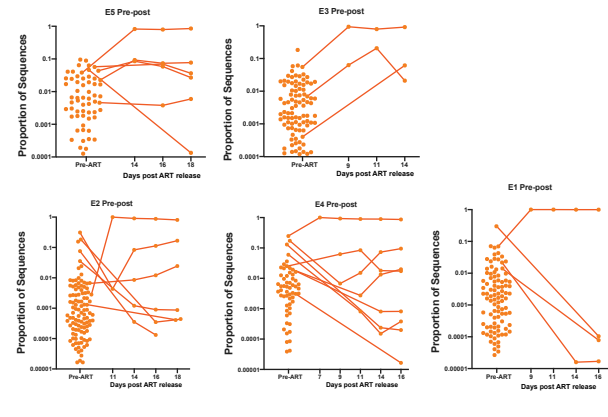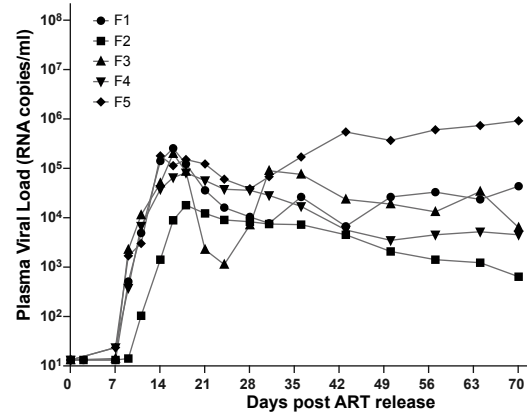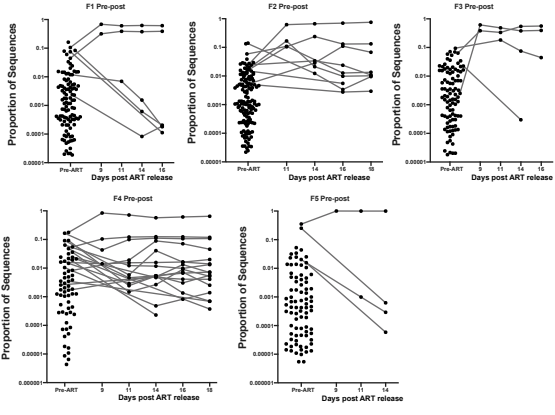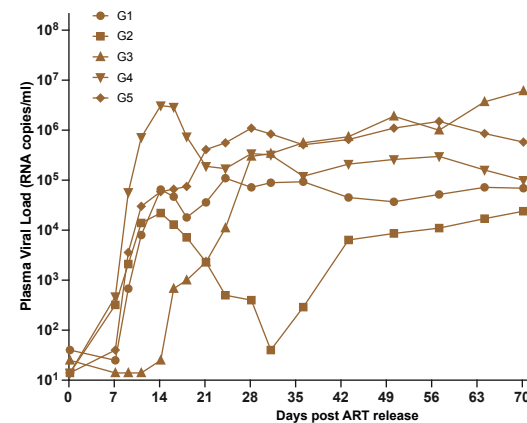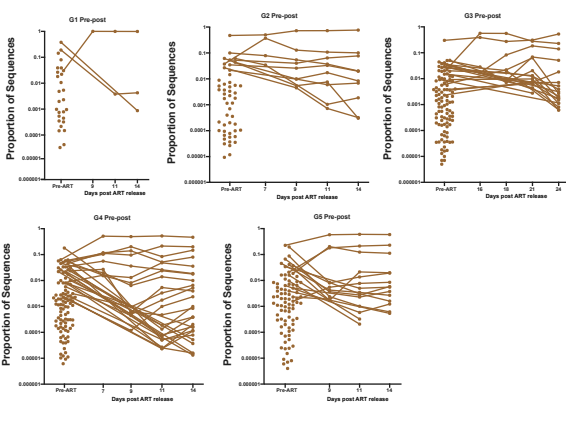

Supplement: S14 Fig — Rebound kinetics and barcodes for each rebounding RM based on day ART initiated (left panel). Barcode comparisons across time post rebound and compared to the pre-ART population (right panel). Groups are color-coded based on days post infection until ART: d6 (purple), d7 (orange), d9 (black) and d12 (brown). (PDF) [file ppat.1012135.s014.pdf]

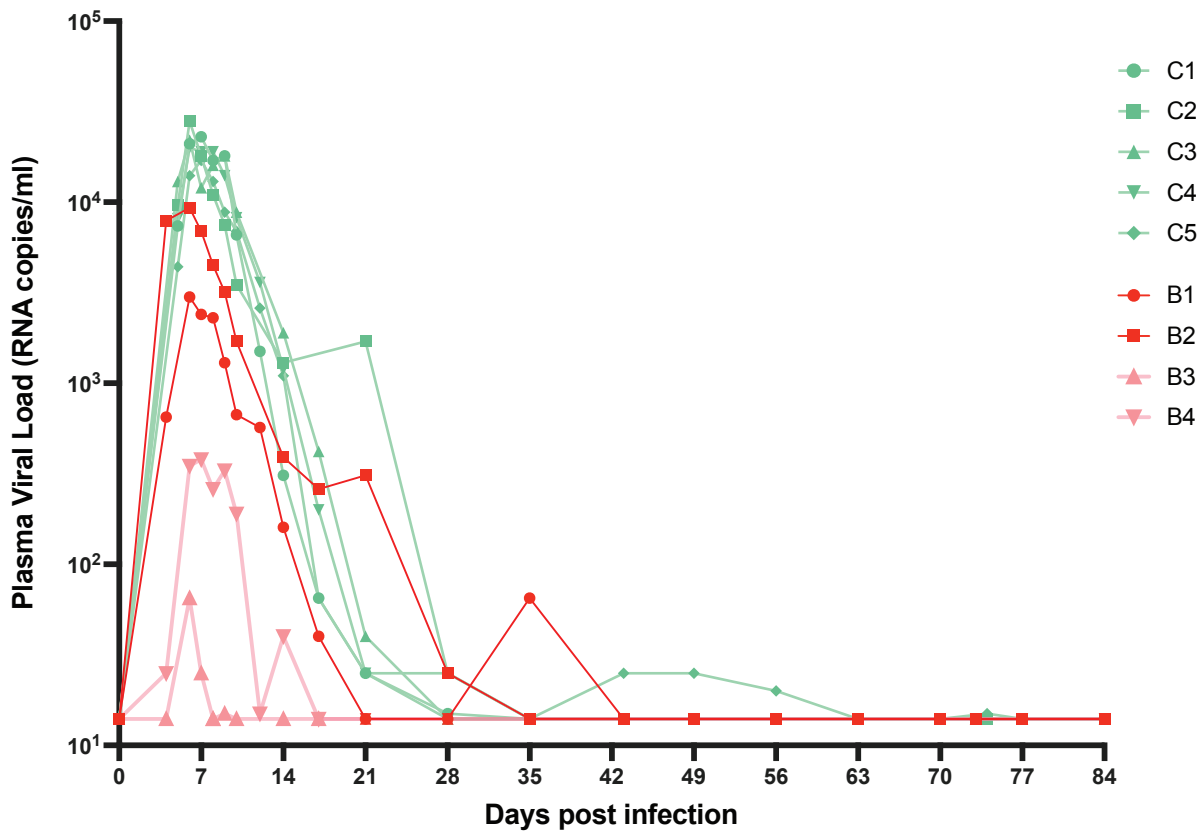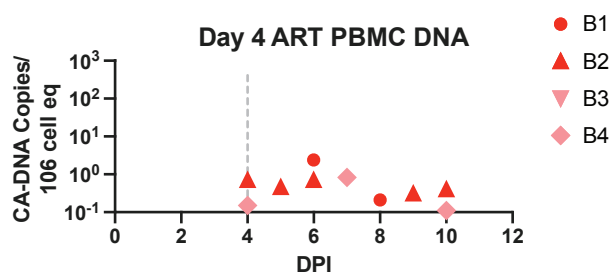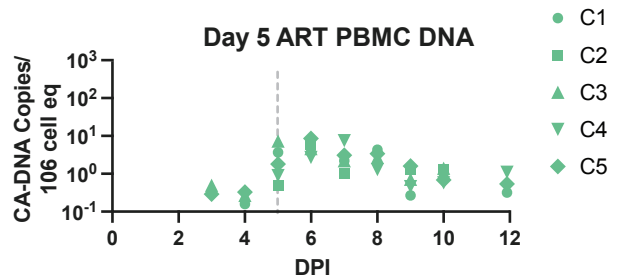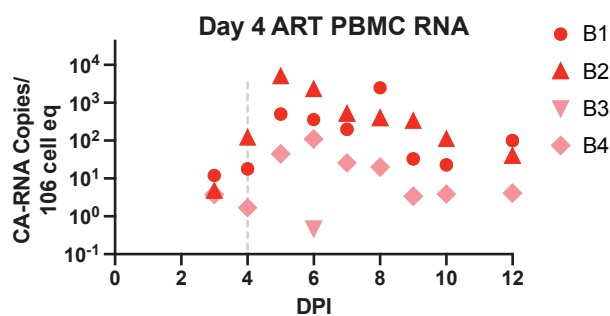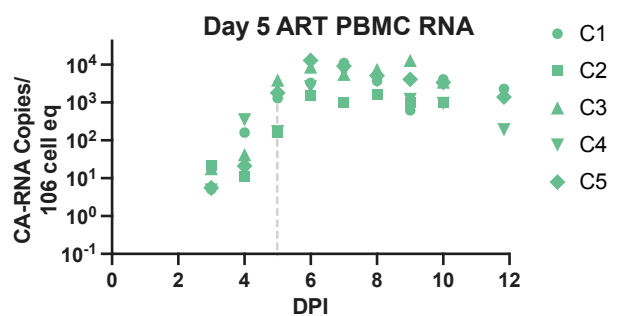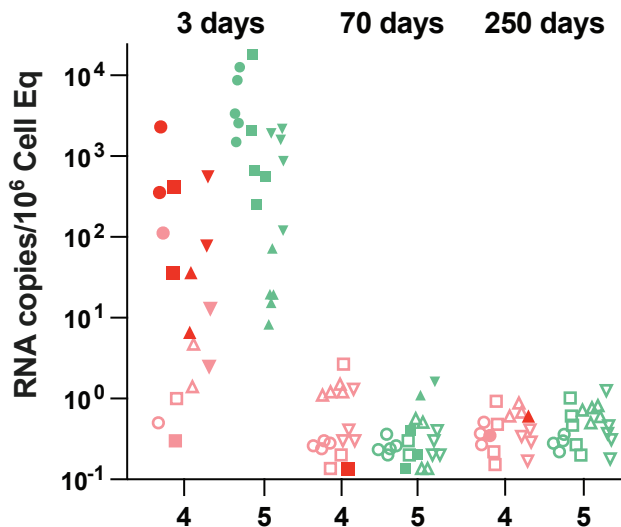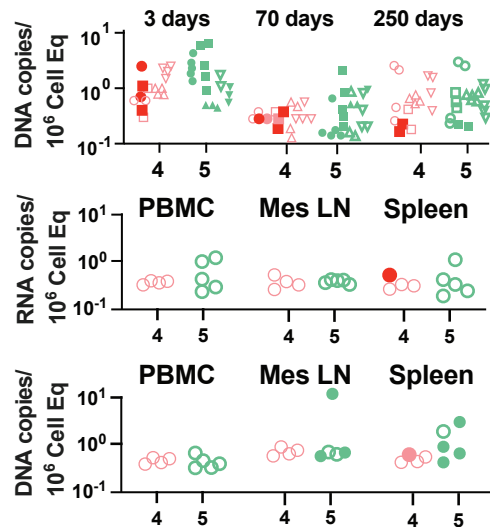

Supplement: S15 Fig — The stochastic nature of RCVR generation and maintenance is highlighted in RMs B1 and B2 (dark red), which failed to rebound despite viral measurements slightly lower than the comparable d5 RMs (green). Decay kinetics of CA-RNA and CA-RNA within PBMC (circle), LNMC (square), DUO (up triangle) and BM (down triangle) from 3-, 70- or 250-days following ART initiation. (PDF) [file ppat.1012135.s015.pdf]
